# Extracellular proteolysis in cancer: Proteases, substrates, and mechanisms in tumor progression and metastasis

**DOI:** 10.1016/j.jbc.2024.107347

**Published:** 2024-05-06

**Authors:** Evette S. Radisky

**Affiliations:** Department of Cancer Biology, Mayo Clinic Comprehensive Cancer Center, Jacksonville, Florida, USA

**Keywords:** proteolysis, protease, serine protease, metalloprotease, cysteine protease, aspartic protease, cathepsin, zymogen, protein protease inhibitor, extracellular matrix, tumor microenvironment, proteolytic signaling, cancer progression, metastasis, degradomics

## Abstract

A vast ensemble of extracellular proteins influences the development and progression of cancer, shaped and reshaped by a complex network of extracellular proteases. These proteases, belonging to the distinct classes of metalloproteases, serine proteases, cysteine proteases, and aspartic proteases, play a critical role in cancer. They often become dysregulated in cancer, with increases in pathological protease activity frequently driven by the loss of normal latency controls, diminished regulation by endogenous protease inhibitors, and changes in localization. Dysregulated proteases accelerate tumor progression and metastasis by degrading protein barriers within the extracellular matrix (ECM), stimulating tumor growth, reactivating dormant tumor cells, facilitating tumor cell escape from immune surveillance, and shifting stromal cells toward cancer-promoting behaviors through the precise proteolysis of specific substrates to alter their functions. These crucial substrates include ECM proteins and proteoglycans, soluble proteins secreted by tumor and stromal cells, and extracellular domains of cell surface proteins, including membrane receptors and adhesion proteins. The complexity of the extracellular protease web presents a significant challenge to untangle. Nevertheless, technological strides in proteomics, chemical biology, and the development of new probes and reagents are enabling progress and advancing our understanding of the pivotal importance of extracellular proteolysis in cancer.

Proteins are highly chemically stable—uncatalyzed hydrolysis of peptide bonds at neutral pH and ambient temperature takes hundreds of years (1). Because the human body is comprised largely of proteins, there is a requirement for efficient proteolysis to dynamically remodel and regulate the proteome to meet ongoing biological needs, to recycle spent proteins into useful building blocks, and to digest protein nutrients. Furthermore, proteomic analyses have revealed that many human proteins undergo proteolytic cleavage to reach their mature forms, making limited proteolysis one of the most prevalent posttranslational modifications (2). Proteases (also known as peptidases or proteinases) are the enzymes that catalyze peptide bond hydrolysis and constitute more than 3% of human protein-encoding genes (2). These highly proficient enzymes can cleave dozens, hundreds, or even thousands of peptide bonds per second, accelerating peptide bond hydrolysis by 10 to 13 orders of magnitude over uncatalyzed rates (1). While essential for normal physiological function, dysregulation of these powerful enzymes has the potential to wreak biological havoc, and indeed proteases also play pathological roles in many diseases, including cancer.

In the extracellular environment of tumors, the interplay of proteases, substrates, and effectors shapes biological and pathological processes crucial to cancer development. This tumor microenvironment includes tumor cells, immune cells, fibroblasts, vascular cells, and adipocytes, all of which communicate through a variety of secreted proteins and direct protein–protein interactions mediated by transmembrane and membrane-associated proteins and protein complexes. These cells are additionally influenced by their interactions with the extracellular matrix (ECM), whose architecture, composition, and stiffness are determined by ECM proteins, glycoproteins, and proteoglycans. Proteases are leading actors on this extracellular stage—by processing secreted, membrane-associated, and ECM proteins, proteases actively remodel the composition of the extracellular proteome. This remodeling involves releasing, activating, and inactivating bioactive proteins and peptides, modulating cell surface receptors and adhesion molecules, and altering the chemical and biophysical nature of the ECM. Not all such actions of proteases in the tumor microenvironment promote disease progression; some extracellular proteases play a role in tumor suppression, while others exhibit both pro- and anti-cancer effects depending on cancer type, stage, site of expression, and the model studied (3, 4). Additionally, a different set of proteases influences cancer development, progression, and response to therapy from inside the cell, impacting intracellular processes including apoptosis (5), gene transcription (6, 7), DNA repair (8, 9), and cellular metabolism (10, 11). Interestingly, some classically extracellular proteases also affect cancer through dual extra- and intracellular localization and hydrolysis of intracellular substrates (12).

The focus of this review is on the extracellular tumor-promoting actions of proteases, encompassing major players, their structures and catalytic mechanisms, their posttranslational regulation and dysregulation in cancer, and the mechanisms through which proteolysis of specific extracellular substrates contributes to tumor growth, progression, and metastasis. Additional topics include current and emerging technologies that are enhancing our ability to link specific proteases to specific substrate cleavage events and to understand the pathological consequences. The emerging picture is complex, involving myriad interconnected proteases, substrates, regulators, and effectors, the continued deciphering of which may lead to effective therapeutic strategies targeting proteases and proteolytic pathways.

## Cancer-promoting extracellular proteases: an ensemble cast of characters

Human extracellular proteases are categorized into four classes based on their chemical mechanisms of catalysis: metalloproteases, serine proteases, cysteine proteases, and aspartic proteases. These classes further branch into evolutionary families. In the following sections, proteases are identified by class and family as annotated within the Merops Peptidase Database (https://www.ebi.ac.uk/merops/). Extracellular proteases from all four classes play roles in cancer progression (Table 1), sharing common functions and regulatory patterns across classes and families. A notable similarity is their pattern of recognizing substrate cleavage sites. The substrate peptide chain binds almost universally in an extended β-strand conformation (13), within a catalytic cleft of the enzyme containing the active site. The substrate amino acid residues upstream of the cleavage site are designated P_1_, P_2_, P_3_, *etc.* toward the protein N-terminus, and downstream of the cleavage site are designated P_1_ʹ, P_2_ʹ, P_3_ʹ, *etc.* towards the protein C-terminus. These residues dock into complementary binding pockets on the enzyme called subsites, correspondingly designated S_1_, S_2_, S_3_, *etc.* for the upstream (non-primed) subsites and S_1_ʹ, S_2_ʹ, S_3_ʹ, *etc.* for the downstream (primed) subsites (14). While specific subsites vary in importance between different protease classes and families, general patterns of recognition remain consistent. The substrate positioning for cleavage involves backbone-to-backbone hydrogen bonds between the substrate and fixed structural elements of the enzyme (the polypeptide binding site), often forming an antiparallel β-sheet pattern of hydrogen bonds within the active site cleft (15). Additionally, proteases across classes recognize specific protein substrates using exosites and auxiliary binding domains, which interact with substrate features further distant from the cleavage site (16, 17). This overview of protease classes and their substrate recognition sets the stage for a more focused examination of specific protease families, beginning with the pivotal role of metalloproteases in cancer progression.

**Table 1:** Selected extracellular proteases with roles in tumor progression and metastasis

Class	MEROPS family	Protease^a^	Alternative names	Selected cancer-relevant extracellular substrates^a^
Metalloproteases	M10	MMP-1	collagenase 1	collagens I, II, III; proTGF-α; proHB-EGF; pro-amphiregulin; PAR1
		MMP-2	gelatinase A	collagens I, IV; fibronectin; elastin; laminin-332; TGFβ LAP; cystatin C
		MMP-3	stromelysin 1	collagens III, IV; laminin; fibronectin; elastin; proteoglycans; VEGF; E-cadherin; proMMP-9; proMMP-1
		MMP-7	matrilysin	collagen IV; elastin; laminin; fibronectin; proteoglycans; VEGF; proHB-EGF; RANKL; E-cadherin
		MMP-9	gelatinase B	collagen IV; fibronectin; elastin; laminin-111; TGFβ LAP; VEGF; IL-8; E-cadherin
		MMP-10	stromelysin 2	collagen III, IV; laminin; fibronectin; proteoglycans; proMMP-1
		MT1-MMP	MMP-14	collagens I, II, III, IV; laminin-332; fibronectin; proteoglycans; thrombospondin-1; TGFβ LAP; LTBP-1; RANKL; EphA2; proMMP-2
		MT2-MMP	MMP-15	collagen IV; proMMP-2
		MT3-MMP	MMP-16	collagens I, II, III, IV
	M12	ADAM-10	alpha-secretase	pro-EGF; RANKL; E-cadherin
		ADAM-15		proHB-EGF; pro-amphiregulin; E-cadherin
		ADAM-17	TACE	proTGF-α; pro-HB-EGF; pro-amphiregulin; desmoglein-2
		ADAMTS-1		aggrecan; versican; proTGF-α; proHB-EGF; pro-amphiregulin
		ADAMTS-4	aggrecanase 1	brevican; aggrecan; versican; biglycan
		ADAMTS-5	aggrecanase 2	brevican; aggrecan; versican; biglycan

Serine proteases	S1	uPA		plasminogen; PDGF-C; PDGF-D; CDCP1; proKLKs
		tPA		plasminogen; PDGF-C
		plasmin		laminin; fibronectin; proteoglycans; LAP; VEGF; IL-8; PAR-4; CDCP1; pro-MMP-1; proMMP-3; pro-uPA; pro-elastase; proKLKs
		thrombin		fibrinogen; IL-8; FGF-2; PAR1; PAR3; PAR4; proKLKs
		trypsin 1	cationic trypsin	fibronectin; laminin; PAR2; pro-uPA
		trypsin 2	anionic trypsin	collagen 1; fibronectin; laminin; PAR2; pro-uPA; proMMP-1, -2, -3, -9, -13, proMT1-MMP; TIMP-1
		mesotrypsin	PRSS3, trypsin IV	PAR1; PAR2; PAR4; CD109; proKLK5; proKLK7; APPI; APLP2; TFPI-1; TFPI-2; HAI-2; bikunin; TFPI-2
		KLK2		PAR2; proKLKs
		KLK3	prostate-specific antigen (PSA)	proKLKs
		KLK4		PAR1; PAR2; proKLKs; pro-uPA
		KLK5		desmoglein-1; proKLKs
		KLK6		PAR2; proADAM-10
		KLK7		E-cadherin; desmoglein-2
		KLK13		laminin
		chymase		proMMPs; LTBP-1
		matriptase	MT-SP1	laminin-332; proHGF; proMSP; PDGF-C; PDGF-D; PAR2; desmoglein-2; pro-matriptase; pro-uPA
		hepsin		laminin-332; fibronectin; proHGF; proMSP; pro-hepsin; pro-uPA
		TMPRSS2		nidogen-1; proHGF; proTMPRSS2; pro-matriptase
		neutrophil elastase	elastase 2; HLE; PMN elastase	elastin; thrombospondin-1; laminin-111; proteoglycans; LTBP-1; proMMP-3; TIMP-1
		cathepsin G		elastin; thrombospondin-1; RANKL; PAR1; proMMP-3; pro-MMP9
	S9	DPP IV	CD26	CXCL9; CXCL10; CXCL11
		FAP	seprase	collagen I

Cysteine proteases	C1	cathepsin B		collagen I, II, IV; laminin; fibronectin; proteoglycans; E-cadherin; pro-cathepsin D; pro-uPA; TIMP-1; TIMP-2
		cathepsin L		collagen I, II, IV; laminin; fibronectin; elastin; proteoglycans; E-cadherin; EphA2; pro-cathepsin D; proheparanase
		cathepsin S		collagen I, II, IV; laminin-332; fibronectin; elastin; nidogen-1 and -2; proteoglycans; E-cadherin; EphA2; JAM-B
	C13	legumain		fibronectin; pro-legumain; pro-cathepsins; proMMP-2

Aspartic proteases	A1	cathepsin D		collagen I, III; fibronectin; plasminogen; proteoglycans; FGF-2; pro-cathepsin B, pro-cathepsin L; cystatin C

a Proteases and substrates listed are representative rather than comprehensive. Further details and references are found within the text.

### Metalloproteases

*Matrix metalloproteinases (MMPs)*, belonging to *protease family M10*, were among the first extracellular proteases recognized for their role in cancer progression due to their involvement in degrading the ECM, a critical step in tumor invasion and metastasis (18, 19). There are 23 MMPs in humans, including 17 secreted and 6 membrane-associated proteases (20). Among these, *Membrane-type 1 MMP* (*MT1-MMP, or MMP-14*), is crucial for the initial stages of invasion by degrading fibrillar collagen (21, 22, 23, 24), while the gelatinases *MMP-2* and *MMP-9,* along with other MMPs, further degrade the resultant collagen fragments and other ECM proteins (25, 26). These functions are further discussed in the sub-section, “*Degradation and remodeling of the extracellular matrix (ECM)*”. MMP functions in cancer progression extend far beyond ECM degradation, as fewer than 20% of demonstrated MMP substrates are ECM proteins (27). MMPs also regulate chemokines, cytokines, cell membrane receptors, and other signaling molecules by cleaving specific sites (28). MMPs activate and release growth factors and pro-angiogenic signaling molecules, as detailed in the sub-section, “*Release, activation, and inactivation of soluble signaling molecules*”. MMPs also facilitate cancer cell invasion and malignancy by cleaving cell-surface adhesion molecules, discussed further in the sub-section “*Cleavage of transmembrane proteins*”. For example, *MMP-3*, *MMP-7*, and *MMP-9* specifically target E-cadherin to promote cancer cell migration and invasion (29, 30, 31, 32).

The MMP family belongs to the larger *metzincin clan* (33), which also encompasses the *adamalysins*, another family of metalloproteases influential in cancer. Adamalysins include the *ADAM* and *ADAMTS* branches of the *protease family M12*. The 13 catalytically active human *ADAMs* (named for their domain structure including a disintegrin and metalloprotease), are transmembrane-anchored sheddases, the best studied of which are *ADAM-10* and *ADAM-17*. These proteases cleave membrane-associated protein substrates to release soluble ectodomains that can initiate cancer-related signaling processes (34). ADAMs trigger pro-tumorigenic processes through the release of cytokines and growth factors, described in the sub-section “*Release, activation, and inactivation of soluble signaling molecules*”, and by cleaving receptors and adhesion molecules, described in the sub-section “*Cleavage of transmembrane proteins*” (35, 36, 37, 38, 39, 40).

The 19 human *ADAMTSs* (a disintegrin and metalloprotease with thrombospondin motifs), while structurally similar to ADAMs, lack a transmembrane domain and are secreted proteases (41). ADAMTSs participate in shaping the ECM by maturing pro-collagens and cleaving proteoglycans including aggrecan, versican, and brevican, releasing angiogenesis-modulating factors; some ADAMTSs also exhibit sheddase activity (41). In the context of cancer, ADAMTSs contribute to angiogenesis and metastasis through their actions on the ECM and their sheddase functions (42, 43, 44, 45, 46, 47).

#### Structure

**Figure 1:**
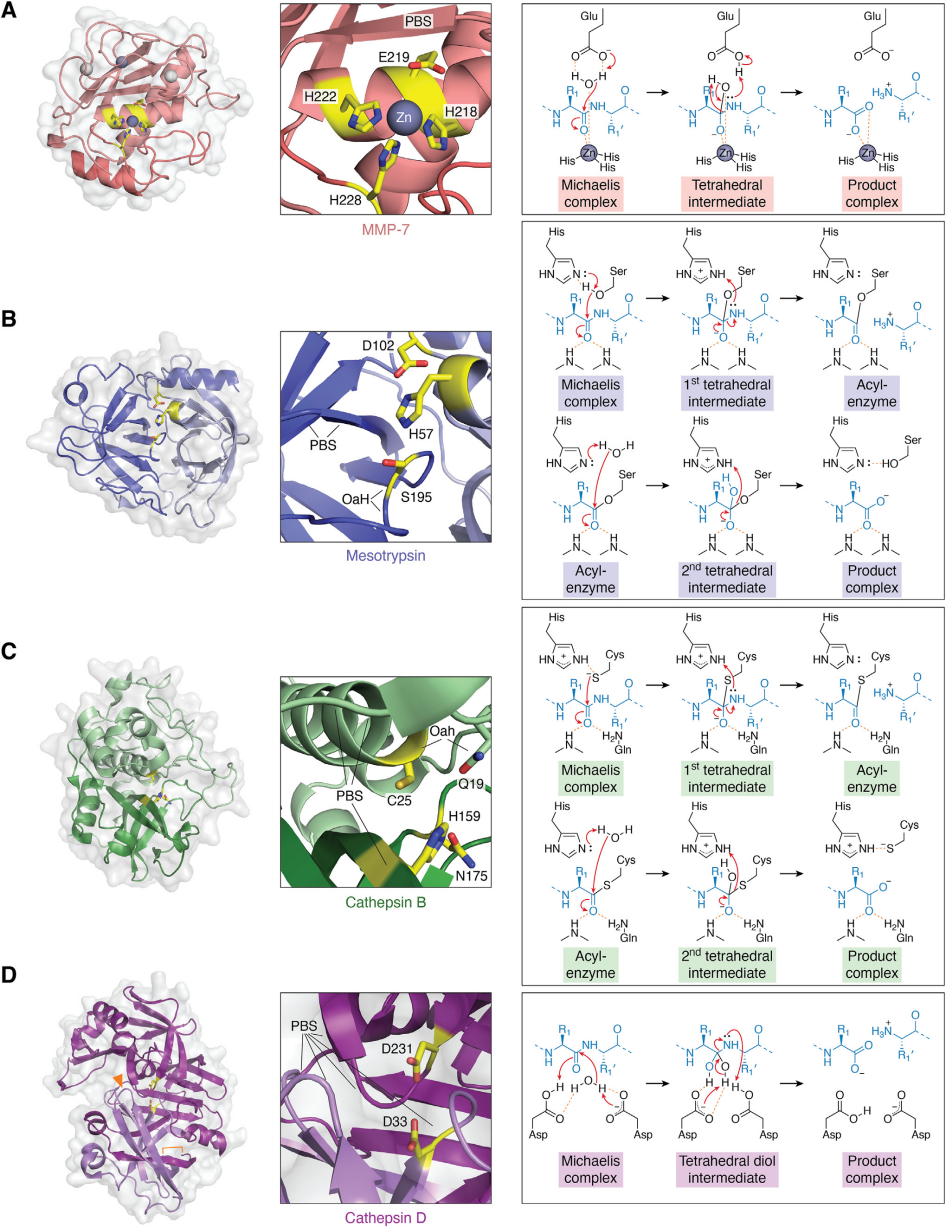
**Protease structures and chemical mechanisms.***A*, the structure of MMP-7 (PDB 1MMQ (48)) is shown as a representative MMP catalytic domain (*left panel*). The domain is divided by a horizontal catalytic cleft separating the *upper* N-terminal subdomain (*light salmon*) and *lower* C-terminal subdomain (*dark salmon*). Substrates bind in the cleft oriented left-to-right in the N- to C-terminal direction, placing the scissile bond near to the catalytic zinc ion (*gray sphere*). Catalytic site detail (*center panel*) shows the catalytic zinc (*gray*) coordinated by three histidine residues and near to a catalytic glutamate residue (*yellow*). The zinc ion and glutamate residue play key roles in catalysis. The β-strand above the catalytic site serves as the substrate polypeptide binding site (PBS). In the conserved catalytic mechanism of MMPs and other metzincins (*right panel*), the catalytic glutamate acts as a base to deprotonate the hydrolytic water, facilitating nucleophilic attack of hydroxide ion on the scissile bond. In the tetrahedral transition state, the oxyanion is stabilized by coordination to the zinc ion. The glutamate subsequently protonates the scissile bond nitrogen to facilitate the cleavage of the peptide bond. *B*, the structure of mesotrypsin (PDB 5TP0 (121)) is shown as a representative S1 serine protease catalytic domain (*left panel*). The domain is comprised of two β-barrels, with the active site located at their interface. The right barrel (*light blue*) contributes catalytic residues His-57 and Asp-102, while the left barrel (*dark blue*) contributes the catalytic Ser-195. Substrates bind in a horizontal cleft oriented left-to-right in the N- to C-terminal direction, placing the scissile bond near the catalytic Ser-195. The catalytic site detail (*center panel*) shows the catalytic triad in *yellow*. Black lines indicate the positions of hydrogen bond participants of the oxyanion hole (OaH) and the polypeptide binding site (PBS). In the conserved catalytic mechanism of serine proteases (*right panel*), histidine acts as a base to deprotonate serine. Serine acts as a nucleophile to attack the scissile bond, leading to the first tetrahedral intermediate, in which the oxyanion is stabilized by hydrogen bonding with backbone amide groups of the oxyanion hole. Histidine then donates a proton to facilitate bond breaking and departure of the amine leaving group, with the formation of the covalent acyl-enzyme intermediate. In the second half of the mechanism, histidine again serves as a base to activate the hydrolytic water molecule, facilitating nucleophilic attack on the acyl-enzyme to form the second tetrahedral intermediate, again stabilized by the oxyanion hole. Finally, histidine donates a proton back to serine in coordination with the release of the carboxylic acid product. *C*, representative of cysteine cathepsins, cathepsin B (PDB 1GMY (159)) is comprised of two domains (*left panel*), with the active site at their interface. By convention, the α-helical domain (*light green*) is referred to as the L domain, and the β-barrel domain (*dark green*) is referred to as the R domain; here, the molecule is rotated to align the substrate binding cleft from left to right as pictured for the other protease families, placing the L domain on top and the R domain beneath. The catalytic site detail (*center panel*) shows the catalytic triad residues Cys-25, His-159, and Asn-175 (papain numbering convention) in *yellow*. Black lines indicate the positions of hydrogen bonding components of the oxyanion hole (OaH) and the polypeptide binding site (PBS). In the conserved catalytic mechanism of C1 cysteine proteases (*right panel*), the Cys-25 thiolate anion attacks the scissile bond, proceeding through a first tetrahedral intermediate which is stabilized by an oxyanion hole. His-159 donates a proton to assist the departure of the amine leaving group, with the formation of the covalent acyl-enzyme intermediate. Subsequently, the acyl-enzyme is hydrolyzed by a nucleophilic water molecule, proceeding through a second tetrahedral intermediate, with His-159 assisting as a base. *D*, the structure of A1 aspartic protease cathepsin D (PDB 1LYB (189)) is comprised of two β-barrel domains separated by a six-stranded β-sheet, with the active site at the domain interface (*left panel*). The mature enzyme is proteolytically processed (site marked by *orange bracket*) to generate the 2-chain enzyme, shown with an N-terminal light chain in *light purple* and a C-terminal heavy chain in *dark purple*. Substrates bind in a cleft, oriented left-to-right, between the domains, placing the scissile bond near the catalytic aspartates. A flexible β-turn “flap” (*orange* arrow) adjusts to enclose the substrate upon binding. The catalytic site detail (*center panel*) shows the two catalytic aspartates in *yellow*. Black lines indicate the positions of hydrogen bonding components of the polypeptide binding site (PBS). In the conserved catalytic mechanism of A1 aspartic proteases (*right panel*), anionic Asp-231 abstracts a proton from the hydrolytic water, while Asp-33 donates a proton to the substrate carbonyl oxygen, generating a *gem*-diol tetrahedral intermediate. The two aspartates subsequently swap their roles as acid and base to facilitate the breaking of the scissile peptide bond.

Metzincins, which include MMPs, ADAMs, and ADAMTSs, share a similar structure in their catalytic domains, characterized by combined α and β folds (33). These proteases often have additional domains for substrate recognition and interaction. MMP-7, structurally the simplest, contains a minimal catalytic domain (48) without extra substrate-binding modules. It forms a compact sphere with a shallow catalytic cleft, dividing it into larger N-terminal and smaller C-terminal subdomains (Fig. 1*A*, left panel). Adamalysins differ from MMPs by having two additional α-helices in their catalytic domains (33). In both MMPs and adamalysins, a conserved sequence at the junction between N- and C-terminal subdomains contains the characteristic conserved sequence H-E-X-X-H-X-X-G-X-X-H (where X is a nonconserved residue). The first seven of these residues form a helix at the back of the active site cleft, with a tight turn at the glycine placing the final histidine near the other two histidines to form a zinc-binding site (Fig. 1*A*, center panel). High-resolution crystal structures of MMPs reveal the active site geometry, including a catalytic water molecule coordinated to both the catalytic zinc ion and the nearby catalytic glutamate residue, and several other water molecules (49).

Substrates bind in the catalytic cleft with the scissile bond near the zinc ion, catalytic water, and catalytic glutamate residue, displacing other water molecules. The substrate peptide backbone aligns *via* hydrogen bonding of P_2_ and P_1_ʹ substrate residues to create an antiparallel β-sheet with the enzyme (50). Substrate specificity arises from the recognition of substrate side chains by enzyme subsites. Most influential is the deep hydrophobic S_1_ʹ subsite formed in part by the long and variable “specificity loop” of the enzyme (33, 50), which typically favors leucine and other bulky hydrophobic residues at the P_1_ʹ position. Another important element of substrate specificity is the shallow hydrophobic S_3_ pocket, which favors proline at the P_3_ position (51). Comprehensive specificity profiling of many family members using high-throughput methods like peptide substrate phage display screening (52) and proteomic screening of human peptide sequences (27) has uncovered more variable substrate preferences among different MMPs at positions P_2_, P_1_, P_2_ʹ, and P_3_ʹ.

#### Catalytic mechanism

Metzincins share a broadly conserved catalytic mechanism (Fig. 1*A*, right panel) (33, 50). The catalytic water molecule becomes polarized by interaction with both the zinc ion and the glutamate residue, which enhances its nucleophilicity. The catalytic glutamate residue abstracts a proton from the water molecule, facilitating attack by the resultant hydroxide ion on the scissile bond, which leads to the formation of a tetrahedral transition state in which the oxyanion is stabilized by coordination with the zinc ion and an auxiliary water molecule (53). In the next phase, the tetrahedral intermediate breaks down, forming the product complex through a series of coordinated proton transfers (50). In the final double-product complex, the newly formed C-terminus interacts with the catalytic zinc, while the newly formed N-terminus engages with the catalytic glutamate. A new water then enters, binds to the zinc ion, and displaces the C-terminal product, leading to further rearrangements in the active site that stimulate the release of the N-terminal product (49, 50).

### Serine proteases

The *S1 serine protease family*, also known as the *chymotrypsin* or *trypsin* family, is the largest single family of human proteases, with 118 characterized and putative enzymes (Mammalian Degradome Database: http://degradome.uniovi.es/dindex.html; (54)). These proteases are mostly secreted or extracellularly oriented transmembrane proteins, although a few perform intracellular proteolytic functions. Many members of this family have been linked to tumor progression and metastasis, including the tumor cell-produced *urokinase-type plasminogen activator (uPA)* and its substrate *plasmin*, involved in the directed degradation of ECM during cellular invasion as well as in activation of the collagen-degrading MMPs (25, 26).

Tumor cells can also activate the serine protease-mediated coagulation cascade, leading to the activation of *thrombin*. Thrombin plays a key role in metastasis by converting fibrinogen into fibrin and activating platelets (55, 56, 57). Activated platelets aggregate with tumor cells, forming cellular complexes that adhere to the endothelium and subendothelial matrix, facilitating extravasation. These processes also contribute to tumor growth, angiogenesis, and escape from immune surveillance (58, 59, 60, 61, 62). Thrombin also cleaves and activates *protease-activated receptors (PARs)*, triggering signal transduction pathways with far-reaching impact on cellular proliferation, survival, and motility (59, 60, 61, 62, 63, 64), as described further in the sub-section “*Cleavage of transmembrane proteins*”. It should be noted that signaling through cleavage of PARs, although elucidated initially for thrombin/PAR1 (65), extends to many other serine proteases (66, 67, 68, 69), and even proteases from other classes (70).

*Trypsins*, another branch of the S1 family, are also implicated in ECM degradation during tumor cell invasion (71), activation of uPA (72) and MMP zymogens (73, 74), and signaling through cleavage of PARs (75, 76). Specifically, the trypsin isoform *PRSS3/mesotrypsin* is noted for its role in several types of cancer, targeting a restricted subset of substrates that include PARs and proteinaceous inhibitors of other serine proteases, as described further in the sub-section, “*Regulation of other enzymes within the protease web – activation of zymogens, degradation of inhibitors*” (77, 78, 79, 80, 81, 82, 83).

The *kallikreins*, a significant branch of the S1 family with 15 secreted enzymes, are often upregulated in different forms of cancer. Some of these proteases serve as biomarkers of cancer progression, including most notably *kallikrein-related peptidase 3* (*KLK3*, also known as *prostate-specific antigen PSA*), and many have also been investigated as potential contributors to cancer progression, reviewed in (84). While direct roles in tumor progression are not fully established for most KLKs, cell culture studies suggest that these enzymes participate in protease activation cascades (85, 86, 87, 88), cleave various ECM proteins and adhesion molecules (88, 89, 90, 91, 92), and signal through PARs (93, 94, 95), potentially influencing tumor growth.

The *type II transmembrane serine proteases (TTSPs)*, another significant branch of the S1 family, include 17 members in humans. Of these, ten have been studied in cancer, acting either as tumor promoters or suppressors, depending on the protease and the context (96). Key TTSPs implicated in cancer development and progression include *matriptase*, *hepsin*, and *TMPRSS2*. These enzymes cleave and activate growth factors, such as hepatocyte growth factor (HGF), triggering signaling pathways leading to tumor growth, progression, and metastasis as described in the sub-section “*Release, activation, and inactivation of soluble signaling molecules*” (97, 98, 99, 100, 101, 102). They also activate other pro-tumorigenic proteases, including uPA (102, 103, 104, 105). Matriptase, specifically, can directly cleave and initiate oncogenic signaling through PAR2, detailed in the sub-section “*Cleavage of transmembrane proteins*” (103, 106).

Secreted neutrophil serine proteases, especially *neutrophil elastase* and *cathepsin G*, contribute to metastasis through ECM degradation and remodeling (107, 108), MMP activation (109), and membrane receptor cleavage (110, 111). Recent evidence suggests that the role of neutrophil elastase in cancer may be more complex than previously thought, as it has been discovered that neutrophil elastase can enter cells through endocytosis and selectively kill malignant cells through cleavage of intracellular death receptor CD95 (112). This new understanding challenges the previously established paradigm that neutrophil elastase only promotes tumor growth and progression. Additional studies are needed to clarify the dual nature of neutrophil elastase in different pathological settings.

The *S9 serine protease family* includes several type-II transmembrane proteases unrelated to the TTSPs. The best studied among these in cancer progression are *dipeptidyl peptidase IV* (*DPP IV*; also known as *CD26*) and *fibroblast activation protein* (*FAP*; also known as *seprase*). DPP IV, located on epithelial cells and lymphocytes, plays a crucial role in cancer by processing peptide hormones, chemokines, and neuropeptides through the proteolytic removal of N-terminal dipeptides. This action can either activate or deactivate these molecules, depending on the specific substrate involved. As a result, DPP IV significantly influences immune system regulation by influencing chemokine signaling. DPP IV also exhibits complex biological activities, both pro- and anti-tumorigenic, in different disease settings (113). Notably, it promotes tumor growth by altering interferon-inducible chemokines like CXCL9, CXCL10, and CXCL11, converting these agonists of receptor CXCR3 to antagonists, and greatly reducing chemotactic potency (114). This activity of DPP IV can suppress host antitumoral immune responses, and DPP IV inhibition has shown promise in enhancing natural tumor immunity and improving responses to immunotherapy, for example in models of hepatocellular carcinoma and melanoma (115, 116). FAP is expressed primarily on the surface of activated fibroblasts and some invasive tumor cells (117, 118). It displays protumorigenic activities in colon and lung cancer models, linked to its role in remodeling, degrading, and clearing collagen fragments (119, 120). Due to its involvement in these processes, FAP has been extensively studied as a potential biomarker for cancer and as a target for imaging agents in cancer diagnosis and treatment.

#### Structure

Members of the S1 serine protease family share a similar composite catalytic domain comprised of two β-barrels, with the active site located at the interface between the barrels, as exemplified by human mesotrypsin (121) (Fig. 1*B*, left panel). Some S1 family proteases have extra accessory domains that help in substrate recognition or perform regulatory functions, enhancing their specificity and functional diversity. Chymotrypsin, elastase, and trypsin were among the first protein structures revealed by X-ray crystallography (122, 123, 124, 125), and trypsin was among the first proteins probed by site-directed mutagenesis (15, 126, 127, 128), contributing to our understanding of their mechanism. All active proteases in the family feature a catalytic triad consisting of serine, histidine, and aspartate; in the chymotrypsin numbering convention, these residues are Ser-195, His-57, and Asp-102 (Fig. 1*B*, center panel).

The substrate binds within a groove crossing the enzyme horizontally, such that the nonprimed substrate residues interact with the enzyme’s left barrel and the primed residues interact with the right barrel. The interaction involves an antiparallel β-sheet pattern of hydrogen bonds between the P_3_ and P_1_ substrate residues and a β-strand of the enzyme, forming the polypeptide binding site (15). The scissile bond of the bound substrate is positioned near the catalytic serine side chain. Substrate specificity is conferred by the interaction of the substrate sidechains with enzyme subsites, with recognition of the P_1_ side chain by the S_1_ specificity pocket playing a dominant role. Many enzymes in this family cleave after lysine or arginine; these proteases characteristically possess an Asp-189 residue at the base of the deep S_1_ specificity pocket, which forms a salt bridge with the bound substrate. Mutagenesis studies have shown that P_1_ specificity depends not only on the enzyme residues that directly contact the substrate but also on several surface loops of the enzyme that maintain the conformational stability of the S_1_ specificity pocket (15, 129, 130). Substrate specificity at more distant subsites is also modulated by multiple surface loops of the enzymes that vary significantly among family members in terms of their length, sequence, and conformation. The dynamics of these surface loops can also regulate enzyme activity and control access to the substrate-binding site (129, 130).

S9 family catalytic domains belong to the α/β hydrolase superfamily, a different fold from the S1 family, and also feature a Ser-His-Asp catalytic triad (131, 132). Proteases including DPP IV and FAP have an additional 8-bladed β propeller domain and require dimerization for activity. The catalytic site is located within a large cleft between the two domains, and both the propeller and catalytic domains contribute to substrate binding and the dimerization interface. The histidine residue of the catalytic triad is situated in a loop that is part of the dimerization interface, accounting for the requirement for dimer formation to stabilize the active site and enhance enzyme activity (132). DPP IV and related family members cleave N-terminal dipeptides specifically after P_1_ proline residues, as favored by the hydrophobic S_1_ subsite within the catalytic domain. The charged amino terminus of the P_2_ residue is positioned *via* salt bridges formed with a double glutamate motif located within an α-helical insertion of the β propeller domain (131, 132). Despite the unique architecture of their catalytic domain, the chemical mechanism of catalysis in S9 family serine proteases is believed to follow a stepwise sequence very similar to that found in the S1 family serine proteases.

#### Catalytic mechanism

S1 family serine proteases share a conserved multistep catalytic mechanism (Fig. 1*B*, right panel) (15). Upon productive binding of the substrate, the catalytic serine is activated as a nucleophile through deprotonation by histidine. The serine hydroxyl then attacks the carbonyl carbon of the scissile peptide bond, leading to the formation of a transient tetrahedral intermediate, which closely resembles the transition state of the reaction. In this intermediate, the negative charge of the oxyanion is stabilized by hydrogen bonds with the backbone amide groups of Ser-195 and Gly-193 (chymotrypsin numbering), a configuration referred to as the oxyanion hole (133). The tetrahedral intermediate then collapses with the departure of the amine leaving group, leading to the formation of the more stable covalent acyl-enzyme. A water molecule enters the active site and becomes activated for nucleophilic attack on the acyl-enzyme, *via* deprotonation by His-57. The nucleophilic attack leads to a second tetrahedral intermediate, again stabilized by the oxyanion hole. This intermediate then collapses, with the Ser-195 hydroxyl group reclaiming a proton from His-57, in coordination with the release of the carboxylic acid product. Many high-resolution crystal structures have validated the molecular details of the serine protease reaction sequence. Atomic resolution structures of trypsin acyl-enzymes with substrates have clearly shown the water molecule involved in hydrolysis (134). While the authentic tetrahedral intermediates for efficient substrates are too unstable to be directly observed, they have been modeled by inhibitor complexes that mimic these intermediates (134, 135) and corroborated by theoretical studies (136). These studies have revealed that His-57 and Ser-195 undergo subtle shifts in orientation during the reaction, facilitating the progression of the catalytic reaction.

There has been considerable debate about the precise role of Asp-102, the third member of the catalytic triad. It is strategically positioned for hydrogen bonding with His-57, and its mutagenesis can slow catalysis by four orders of magnitude (126). Initially, it was proposed that Asp-102 participates in a charge relay system by accepting a proton from His-57 (137, 138). A subsequent concept proposed that His-57 and Asp-102 may form a strong, low-barrier hydrogen bond, sharing a proton to enhance the nucleophilicity of Ser-195 (139). A more recently proposed theory, aligning with NMR data on the p*K*_a_ values of these residues, suggests that ionized Asp-102 stabilizes the charge on His-57 in the tetrahedral intermediate and properly orients His-57 with the correct tautomer to function as a base in relation to Ser-195 and the substrate (15, 126). This alternate model gained support from studies on α-lytic protease, a bacterial homolog of the mammalian S1 family proteases. A sub-angstrom crystal structure of α-lytic protease bound to a peptidyl boronic acid, mimicking the transition state, placed the proton on an ionized His-57 hydrogen bonded to an ionized Asp-102 (135). Additionally, a multinuclear 3D-NMR study showed that the p*K*_a_ of Asp-102 is below 1.5, inconsistent with the matched p*K*_a_ values that would be required for a low-barrier hydrogen bond (140), further supporting this alternative model. The current consensus on the catalytic mechanism is that a network of ordinary hydrogen bonds, including the His-57 – Asp-102 interaction, cooperatively positions the catalytic triad and substrate for reaction and stabilizes the oxyanion transition state (15, 135, 141). The importance of this network of remote interactions, which becomes most optimized in the transition state, is also supported by observations from many S1 family proteases that substrate discrimination is largely due to faster cleavage rates of good substrates (higher *k*_cat_), rather than differences in substrate binding affinity (*K*_m_) (15).

### Cysteine proteases

Cathepsins are a group of proteases functioning under acidic conditions within the lysosome and do not belong to a single mechanistic class or evolutionary family, but instead include serine proteases (cathepsins A and G), aspartic proteases (cathepsins D and E), and 11 human proteases of the *C1 cysteine protease family*, which are related to the plant enzyme papain (142). C*ysteine cathepsins*, beyond their vital roles inside cells, can also be secreted into the extracellular space (143), a characteristic often observed in cancer cells. This secretion also plays a crucial role whereby certain immune cells, particularly macrophages, affect the tumor microenvironment (144, 145). Like MMPs, cysteine cathepsins were initially recognized for their roles in cancer progression through their ability to degrade many different ECM proteins, contributing to tumor invasion, metastasis, and angiogenesis (146, 147, 148). Broad profiling of the degradomes of cathepsins B, H, L, S, and Z in a murine pancreatic neuroendocrine tumor model supports general protein degradation as the dominant functional role of cathepsins in this cancer (149). These and other studies have also revealed additional roles for cathepsins, including the targeted proteolytic processing of specific substrates, which can have protumorigenic effects in different cancer contexts (143, 150). Examples of these additional roles include the ectodomain shedding of cell surface adhesion molecules (151, 152, 153), the activation of pro-enzymes through proteolysis (154), and the release of bioactive protein fragments derived from the ECM (155). *Legumain*, a distinct cysteine protease of *family C13*, is another lysosomal enzyme frequently upregulated in cancer. It also can be secreted into the extracellular space, where it can be associated with ECM or with the cell surface (156). Outside the cell, legumain appears to promote metastasis through nonproteolytic signaling mediated through binding to integrins (157). While experiments have demonstrated legumain’s enzymatic activity in cancer (158), there is limited evidence for the role of its extracellular proteolytic function in promoting cancer phenotypes.

#### Structure

The cysteine cathepsins adopt the papain protein fold comprising two domains, one primarily α-helical and the other a β-barrel with short helical segments, with the active site located at the domain interface. The structure of human cathepsin B (159) illustrates this arrangement (Fig. 1*C*, left panel). Cathepsin B was the first cysteine cathepsin to be structurally characterized (160) and remains the most thoroughly studied. The catalytic triad consists of Cys-25 from the α-helical domain and His-159 and Asn-175 from the β-barrel domain (papain numbering system; Fig. 1*C*, center panel). The extended polypeptide chain of a substrate binds in a groove crossing the enzyme, with substrate side chains contacting alternately with the α-helical and β-barrel domains (142). Most cysteine cathepsins are endopeptidases, but some also possess amino- or carboxyterminal exopeptidase activity. Cathepsin B, for example, acts as both an endopeptidase and a carboxydipeptidase, with the latter activity conferred by the interaction of the substrate C-terminal P_2_ʹ residue with an occluding loop in the α-helical domain (142, 160). Cysteine cathepsins use an antiparallel pattern of hydrogen bonds for generic substrate recognition. A short loop from the α-helical domain bonds with the substrate P_2_ main chain, and a β-barrel strand bonds with the substrate P_1_ main chain (159, 161) (Fig. 1*C*, center panel). The primary determinant of substrate specificity is the S_2_ pocket, formed by interacting residues on both domains, typically preferring small hydrophobic or aromatic P_2_ residues (142, 150). The specificity of cysteine cathepsins is further influenced by the S_1_ and S_1_ʹ sites, formed by the α-helical and β-barrel domains, respectively (142, 150). While subsites more distant from the cleavage site have a lesser impact on substrate specificity, proteomic profiling has shown that cathepsin B exhibits a strong preference for glycine at the P_3_ʹ position, attributable to steric restrictions imposed by the occluding loop (162).

#### Catalytic mechanism

C1 family proteases, including cysteine cathepsins, follow a catalytic mechanism similar to papain (Fig. 1*C*, right panel) (163). Unlike the serine proteases where substrate binding triggers the deprotonation of the serine nucleophile, papain-like enzymes have a pre-formed nucleophilic thiolate-imidazolium ion pair, formed by catalytic residues Cys-25 and His-159 (papain numbering), in the pH range of 4 to 8 where the enzymes are active (164, 165, 166). A computational study supports the stability of the thiolate-imidazolium ion pair in the resting state of human cathepsin B (167). The third residue in the catalytic triad, Asn-175, also significantly contributes to the catalytic efficiency (*k*_cat_/*K*_m_) of papain (168) and human cathepsin S (169), as shown by mutagenesis studies. This contribution, which mainly affects the catalytic rate constant (*k*_cat_), suggests that Asn-175 plays a key role in stabilizing the thiolate-imidazolium ion pair. Catalysis is initiated by nucleophilic attack on the scissile peptide bond by the thiolate anion, leading to a covalent tetrahedral transition state or short-lived intermediate, followed by formation of an acyl-enzyme intermediate, with His-159 contributing a proton to assist leaving group departure (Fig. 1*C*, right panel). Subsequently, the acyl enzyme is hydrolyzed by a nucleophilic water molecule, with His-159 assisting as a base. The oxyanion in both the transition state and tetrahedral intermediate is stabilized by an oxyanion hole formed by the backbone amide of Cys-25 and the side chain amide of Gln-19 (Fig. 1*C*, center panel). While computational studies have provided conflicting results on the involvement of the hypothetical tetrahedral intermediate in the papain reaction mechanism (170, 171, 172), experimental evidence strongly supports its existence. Mutagenesis studies on papain showed that conversion of Gln-19 to alanine or serine decreased *k*_cat_/*K*_m_ by 60-600-fold, primarily by decreasing the catalytic rate constant *k*_cat_ (173), highlighting the importance of this residue in stabilizing the transition state. Additionally, the crystal structure of papain, covalently inhibited by the transition state analog leupeptin, revealed the tetrahedral oxyanion to be hydrogen bonded in the oxyanion hole (161). Further, a computational study of the human cathepsin K reaction mechanism reinforces the importance of the conserved oxyanion hole in stabilizing both the transition state and the tetrahedral intermediate in cysteine cathepsins (174).

### Aspartic proteases

*Aspartic cathepsins*, like cysteine cathepsins, are primarily lysosomal enzymes that under some conditions can be extracellularly secreted. *Cathepsin D* is notable for its regulated secretion during physiological processes including lactation and mammary gland involution; it is also overexpressed and constitutively secreted by breast and some other cancers (175). Cathepsin D has been implicated in multiple aspects of cancer development and progression, including proliferation, invasion, metastasis, angiogenesis, and resistance to apoptosis (176, 177, 178, 179). The influence of cathepsin D in cancer is partly due to intracellular actions and nonproteolytic signaling outside cells (177, 179, 180, 181). Notably, the secreted enzyme is mostly present in activated form (182), and some cancer-promoting actions involve extracellular proteolysis, such as degrading ECM components and releasing ECM-sequestered fibroblast growth factor 2 (183, 184). Furthermore, the co-localization of cathepsin D with other secreted proteases in the tumor microenvironment can regulate and amplify their tumorigenic activities. For example, activation of pro-cathepsin B secreted by a colorectal cancer cell line was found to be mediated by cathepsin D (185), and cathepsin D can also upregulate cysteine cathepsin activity through degradation of cystatin C, a potent natural inhibitor of cysteine cathepsins (186). In an interesting reciprocal relationship, cysteine cathepsins including cathepsin B are also the major proteolytic activators of cathepsin D in the lysosome (187), and potentially also in the extracellular environment.

#### Structure

Aspartic cathepsins, which include cathepsin D, are part of the *A1 aspartic protease family* and adopt a pepsin-like fold. This structure consists of two similar β-barrel domains and a six-stranded anti-parallel β-sheet between them (188, 189) (Fig. 1*D*, left panel). In active cathepsin D, the N-terminal β-barrel domain is cleaved within a surface loop, creating the two-chain form. Like cysteine and serine proteases, the active site is located at the domain interface, with Asp-33 from the N-terminal domain and Asp-231 from the C-terminal domain forming the catalytic dyad (Fig. 1*D*, center panel). The extended polypeptide chain of a bound substrate lies in a groove between the domains, crossing the enzyme from left to right. Cathepsin D features an extensive polypeptide binding site in which multiple segments of both domains form backbone-to-backbone hydrogen bonds with the P_3_ – P_2_ʹ residues of a bound substrate (188, 189) (as indicated in Fig. 1*D*, center panel). The scissile bond of the bound substrate is positioned near the catalytic aspartate residues. Cathepsin D favors large hydrophobic residues in the P_1_ and P_1_ʹ positions and accommodates bulky P_2_ and P_4_ residues in its broad catalytic cleft (188, 189). A flexible β-turn, or “flap”, within the N-terminal domain adjusts slightly to enclose the substrate upon binding (189).

#### Catalytic mechanism

Based on homology to other A1 family aspartic proteases, cathepsin D and E likely follow a catalytic mechanism similar to that of mammalian pepsin and several related fungal proteases (190) (Fig. 1*D*, right panel). In the free enzyme, Asp-33 is protonated and Asp-231 is charged, with a hydrolytic water molecule positioned between them. When a substrate binds, the water becomes polarized for nucleophilic attack on the carbonyl carbon of the scissile peptide bond. Asp-231 accepts a proton from the hydrolytic water, while Asp-33 donates a proton to the substrate carbonyl oxygen, leading to the formation of a *gem*-diol tetrahedral intermediate. This mechanism aligns with high-resolution X-ray and neutron crystal structures of aspartic proteases complexed with inhibitors that form a *gem*-diol, mimicking the tetrahedral intermediate and transition state, and revealing precise bond lengths and the positions of key protons (190, 191, 192). Subsequently, breakage of the scissile peptide bond is assisted by the donation of a proton to the leaving amino group by Asp-231 or water, along with the recapture of a proton by Asp-33 to regenerate the active enzyme configuration. Comparisons of dozens of crystal structures of aspartic proteases have revealed a network of additional conserved residues and an additional water molecule that hydrogen bond to the catalytic aspartate residues and to the substrate, regulating the protonation states of the catalytic dyad during the catalytic cycle (193). Recent computational studies have further proposed a role for n → π∗ interactions in which developing negative charge on the diol oxygens is donated to carbonyl acceptors within the substrate P_1_ʹ residue and a conserved glycine of the protease, sequentially facilitating nucleophilic attack by water and peptide bond scission (194, 195). The n → π∗ donation mechanism is primarily supported by high-resolution structures of HIV protease, a member of the A2 family of viral aspartic proteases. Similar active site geometry is observed in structures of A1 family mammalian proteases such as renin and β-secretase, supporting the broader applicability of this mechanism across different aspartic protease families.

## The making of villains—posttranslational regulation and dysregulation of proteases

The extracellular proteases described earlier play vital roles in essential biological functions through regulated proteolysis of protein substrates. However, these same actions can become problematic when they are dysregulated in the context of cancer. One form of dysregulation is transcriptional upregulation, which occurs widely for protease genes in cancers (196), although not the focus of this review. Once translated into protein, proteases undergo further regulatory processes which are also widely dysregulated in cancer, resulting in increased proteolytic activity and increased proteolysis of particular substrates with pathological outcomes.

### Maintenance of latency and proteolytic activation

**Figure 2:**
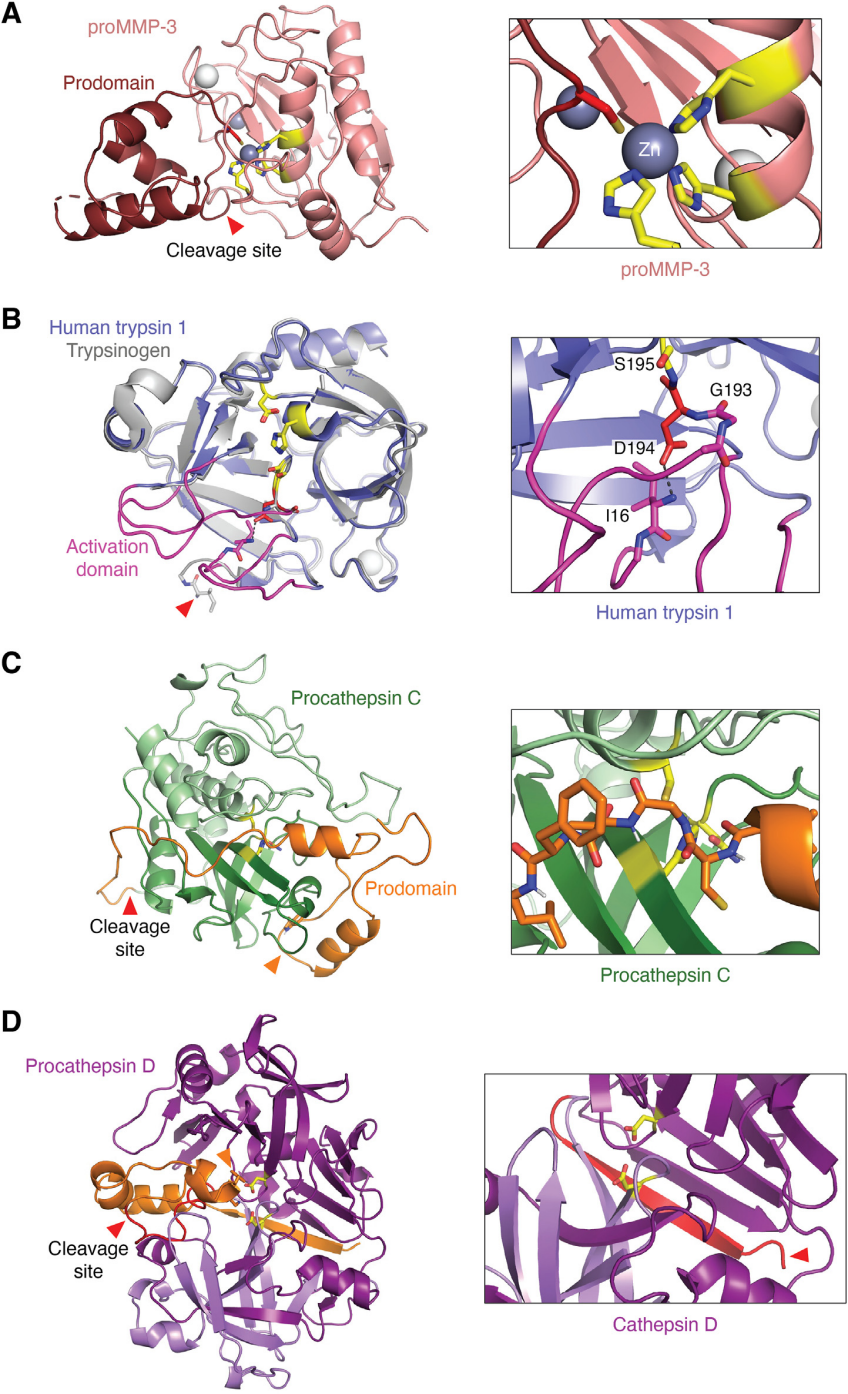
**Protease mechanisms of latency.***A*, the structure of the proMMP-3 catalytic domain (*salmon*; PDB 1SLM (421)) exemplifies the conserved mechanism of metallopeptidase latency, in which the active site is shielded by a prodomain (*brown*) comprised of three helices (*left panel*). Upon cleavage between the catalytic domain and prodomain (cleavage site indicated by the *red arrow*), the prodomain dissociates and the enzyme becomes activated. In the zymogen state, the cysteine residue of the cysteine switch (*red*) coordinates to the catalytic zinc (*gray sphere*), displacing the hydrolytic water molecule (*right panel*). *B*, the crystal structure of human trypsin 1 (*periwinkle*; PDB 2RA3 (422)) superimposed with the structure of bovine trypsinogen (*gray*, *left panel*; PDB 2TGT (423)) illustrates the conserved mechanism of S1 serine protease latency and activation. The zymogen and active forms show few significant differences aside from the untucked N-terminus in the zymogen form (*red arrow*), and the observation that loops comprising the activation domain (*magenta*) are unstructured (and thus invisible in the crystal structure) in the zymogen state. Upon cleavage to remove the propeptide, the new N-terminal residue Ile-16 tucks into the catalytic domain to form a salt bridge (*black dashed line*) between the protein chain N-terminal amine and the Asp-194 side chain (*right panel*). Formation of this salt bridge brings structure to the oxyanion hole by orienting the amide nitrogens of Gly-193 and Ser-195, and shapes the S_1_ specificity pocket to confer catalytic activity. *C*, the structure of procathepsin C (PDB 3PBH (424)) illustrates how the prodomain (*orange*) binds and inhibits the catalytic domain (*green*). The prodomain N-terminal segment contains two helices and forms hydrophobic interactions with the “prosegment binding loop” (*left panel*, *orange arrow*). An extended stretch of prodomain residues crosses the active site (*catalytic triad in yellow*), occupying the substrate-binding cleft in reverse orientation to a true substrate (*detail in right panel*). Low pH disrupts the prodomain structure and electrostatic interactions with the catalytic domain to facilitate proteolytic cleavage at a site indicated by the *red arrow, left panel*, and release of the prodomain. *D*, a homology model of human procathepsin D (*left panel*) illustrates how the prodomain (*orange*) binds and inhibits the catalytic domains (*purple*). The N-terminal segment of the prodomain forms a β-strand interacting with an exosite on the backside of the molecule, while the C-terminal segment of the prodomain covers the active site and inserts a Lys-Tyr anchor (*orange arrow*) to interact with the catalytic Asp residues (*yellow*). Activation involves dissociation of the prodomain and cleavage at a site indicated by the *red arrow*. In mature cathepsin D (*right panel*; PDB 1LYB (189)), the newly generated N-terminus (*red arrow*) undergoes a conformational change to form β-sheet interactions at the exosite vacated by the propeptide. Homology model was generated using Swiss-Model based on the structure of pepsinogen (PDB 2PSG (425)).

Proteases are typically produced as inactive precursors, or zymogens, with limited catalytic activity. They attain full activity through proteolytic cleavage and removal of an N-terminal propeptide or prodomain, which then reveals the fully active enzyme (197, 198). In the case of the MMP family (M10), the N-terminal prodomain is a small globular domain that sterically shields the active site (Fig. 2*A*, left panel). An extended loop of the prodomain fills the substrate-binding cleft of the catalytic domain in an opposite orientation to a true substrate. This loop contains the conserved “cysteine switch” motif P-R-C-G-X-P-D (199), in which the central cysteine residue coordinates to the catalytic zinc ion, displacing the catalytic water molecule and maintaining the zymogen’s latency (200) (Fig. 2*A*, right panel).

MMPs become activated through cleavage by an activating protease, releasing the prodomain. While the specific activating proteases vary by MMP and biological setting, known activators include other active MMPs and secreted serine proteases such as trypsin, plasmin, and mast cell chymases (201). Some MMPs, particularly the membrane-type MMPs, are activated by furin or other proprotein convertases in the secretory pathway before reaching the cell surface (202). Biophysical studies suggest that proteolytic activation begins with conformational changes in the zymogen induced by interactions with the activating protease, leading to the dissociation of the cysteine switch from the zinc ion and the gain of activity. Proteolytic cleavage of the prodomain then renders the activation permanent (203). For some MMPs, full activation may require multiple cleavage events, possibly involving a partially activated intermediate form of the MMP itself (204).

Similar to the membrane-type MMPs, ADAMs, and ADAMTSs from the M12 family typically undergo proteolytic activation within the secretory pathway by furin or other proprotein convertases, which cleave at multiple sites to mature the fully activated enzymes (205). Some ADAMs also use autoproteolytic mechanisms for activation (206). The prodomains of some M12 family members feature a functional cysteine switch as seen in MMPs (207) while in others, the conserved cysteine may bind to the catalytic zinc but is not essential for inhibition by the prodomain (208, 209).

S1 family serine proteases are produced as zymogens with N-terminal extensions of the catalytic domain. These extensions can range from short propeptides to large multi-domain pro-segments. The latency of these proteases is maintained by the deformation and lack of stable conformation in the “activation domain”, which comprises four loops of the catalytic domain that border the active site (Fig. 2*B*, left panel) (197, 210, 211). This deformed or unstructured region includes the S_1_ specificity pocket and the Gly-193 amide nitrogen of the oxyanion hole (chymotrypsin numbering convention). Upon activation through proteolytic cleavage, the newly formed amino terminus folds into the catalytic domain, creating a salt bridge with the buried Asp-194 (Fig. 2*B*, right panel). This conformational change results in the restructuring of the S_1_ specificity pocket and correct orientation of the hydrogen bond donors of the oxyanion hole, leading to full catalytic activity. Depending on the specific protease, the activating cleavage may be autocatalytic, mediated by another extracellular protease (often another S1 family member), or occur within the secretory pathway by furin or another proprotein convertase.

Many serine proteases are activated in physiological cascades, such as the sequential activation of coagulation factors leading to activation of thrombin, the activation of plasmin by plasminogen activators, or the enteropeptidase-mediated activation of trypsin, which then activates other digestive proteases (198). While the specific activators of individual KLKs are not fully defined *in vivo*, biochemical studies implicate a network of reciprocal activating cleavages within this S1 family branch (85). There is also potential for KLK activation by uPA, plasmin, and thrombin (212). The TTSPs hepsin and matriptase are known to autoactivate on the cell surface (213), while TMPRSS2 is autoactivated within the secretory pathway (214). In transgenic mouse models, matriptase autoactivation requires complex formation with the membrane serine protease prostasin as a nonenzymatic cofactor (215), although studies in human cell lines with endogenous matriptase expression suggest that the requirement for prostasin may not be universal (216).

Similar to metalloproteases, cysteine cathepsins are maintained as inactive zymogens by an N-terminal prodomain that physically blocks the active site. The prodomain of these enzymes interacts with two distinct regions: an N-terminal segment forms hydrophobic interactions with an exosite (the “prosegment binding loop”) on the surface of the catalytic domain, and an extended C-terminal stretch occupies the substrate-binding cleft in reverse orientation to a true substrate (Fig. 2*C*) (217, 218). Cysteine cathepsins are activated through an autocatalytic process initiated by the low pH within the lysosome. This acidic environment leads to pH-dependent destabilization of salt bridges and electrostatic interactions, consequently weakening the structural integrity of the prodomain and its interactions with the catalytic domain (197, 219). This process ultimately leads to dissociation of the prodomain and autocatalytic cleavage by the cysteine cathepsin.

Aspartic proteases, including cathepsin D, are kept in a latent state by an N-terminal propeptide that docks at two sites on the catalytic unit to physically block access to the active site. Although the crystal structure of mammalian cathepsin D zymogen has not been reported, insights into its mechanism of latency come from biochemical studies of cathepsin D and crystal structures of similar zymogens like pepsinogen and progastricsin. The latency involves an extended 14-residue N-terminal segment forming a high-affinity interaction with an exosite, while a C-terminal segment covers the active site. This segment inserts side chains of a conserved Lys-Tyr anchor to form a salt bridge with the catalytic aspartate residues (Fig. 2*D*, left panel) (197, 219). As in the cysteine cathepsins, these interactions are disrupted at low pH, leading to enzyme activation and rendering the propeptide susceptible to cleavage. Under acidic conditions, procathepsin D can self-process to remove much of the prodomain, generating active pseudo-cathepsin D. *In vivo*, however, the primary mechanism for activation and maturation of cathepsin D appears to involve a series of cleavages by the cysteine cathepsins B and L in endosomes and lysosomes (187, 220).

In mature cathepsin D, the new N-terminus undergoes a conformational change upon processing and binds to the exosite vacated by the propeptide (Fig. 2*D*, right panel). Interestingly, this conformation is stable only at low pH. At higher pH, the N-terminus dissociates from the exosite and can block the active site cleft, inhibiting the enzyme (221). For an insect cathepsin D homolog, it has been shown that a propeptide cleavage product can re-bind to the exosite at neutral pH, allosterically inhibiting the enzyme by shifting its conformation toward an autoinhibited state (222). It is yet to be determined whether similar regulation by the processed prodomain occurs in human cathepsin D.

Environmental factors play a significant role in modifying and regulating the rates of proteolytic activation of zymogens, including localization to specific sites or complexes, cofactor binding, and pH. These mechanisms can become dysregulated in cancer. Since most proteases are activated through cleavage by other proteases, the entire proteolytic network forms a complex web where the increased activity of one protease, caused by increased abundance or increased activation, can lead to the activation of additional proteases (223).

Activation rates can be regulated and potentially dysregulated through the colocalization of active proteases with their target zymogens. For example, proMMP-2 activation involves a unique cell surface complex that includes an MT1-MMP dimer and the inhibitor TIMP-2. In this complex, TIMP-2 binds to one MT1-MMP molecule in an inhibitory manner, while its non-inhibitory C-terminal domain binds to an exosite on the hemopexin-like domain of proMMP-2, positioning proMMP2 for activation by the second, uninhibited MT1-MMP molecule (224, 225). In invasive thyroid cancers, increased expression of MT1-MMP and its colocalization with proMMP-2 are associated with increased activation and activity of MMP-2 and the development of lymph node metastases (226).

Macromolecular cofactors, such as glycosaminoglycans (GAGs), can play an important role in regulating zymogen activation for many proteases. GAGs, which are oligosaccharide chains that decorate proteoglycans found on cell surfaces and throughout the extracellular matrix, can trigger conformational changes that accelerate autoactivation. For example, GAG binding to the prodomain of cathepsin B accelerates its autoactivation (227). Highly sulfated GAGs also accelerate MMP-7 activation by tethering multiple zymogen molecules along long oligosaccharide chains, facilitating proteolytic interaction among neighboring molecules (228, 229, 230). Similar allosteric and scaffolding effects may be at work among the many other proteases for which zymogen activation is stimulated by GAGs (229).

Another environmental factor influencing zymogen activation is pH. As described above, cysteine and aspartic cathepsins are triggered to activate at the low pH of endosomes and lysosomes. Extracellular acidosis, common in the tumor microenvironment, can also stimulate protease activation. Acidosis has been shown to enhance the autoactivation and shedding of matriptase in a wide variety of epithelial and carcinoma cell models (231, 232). Acidosis can also impact protease activation and proteolysis indirectly, through altered trafficking of lysosomes, cell surface association and secretion of lysosomal proteases, and altered localization and secretion of membrane-associated proteases (233).

In cancer, protease activation cascades and networks can be broadly dysregulated due to changes in protease expression and localization. Thus, protease zymogens are crucial substrates through which many proteases amplify their tumor-promoting effects. This concept is further explored in the sub-section “*Regulation of other enzymes within the protease web – activation of zymogens, degradation of inhibitors*”.

### Regulation by endogenous inhibitors

**Figure 3:**
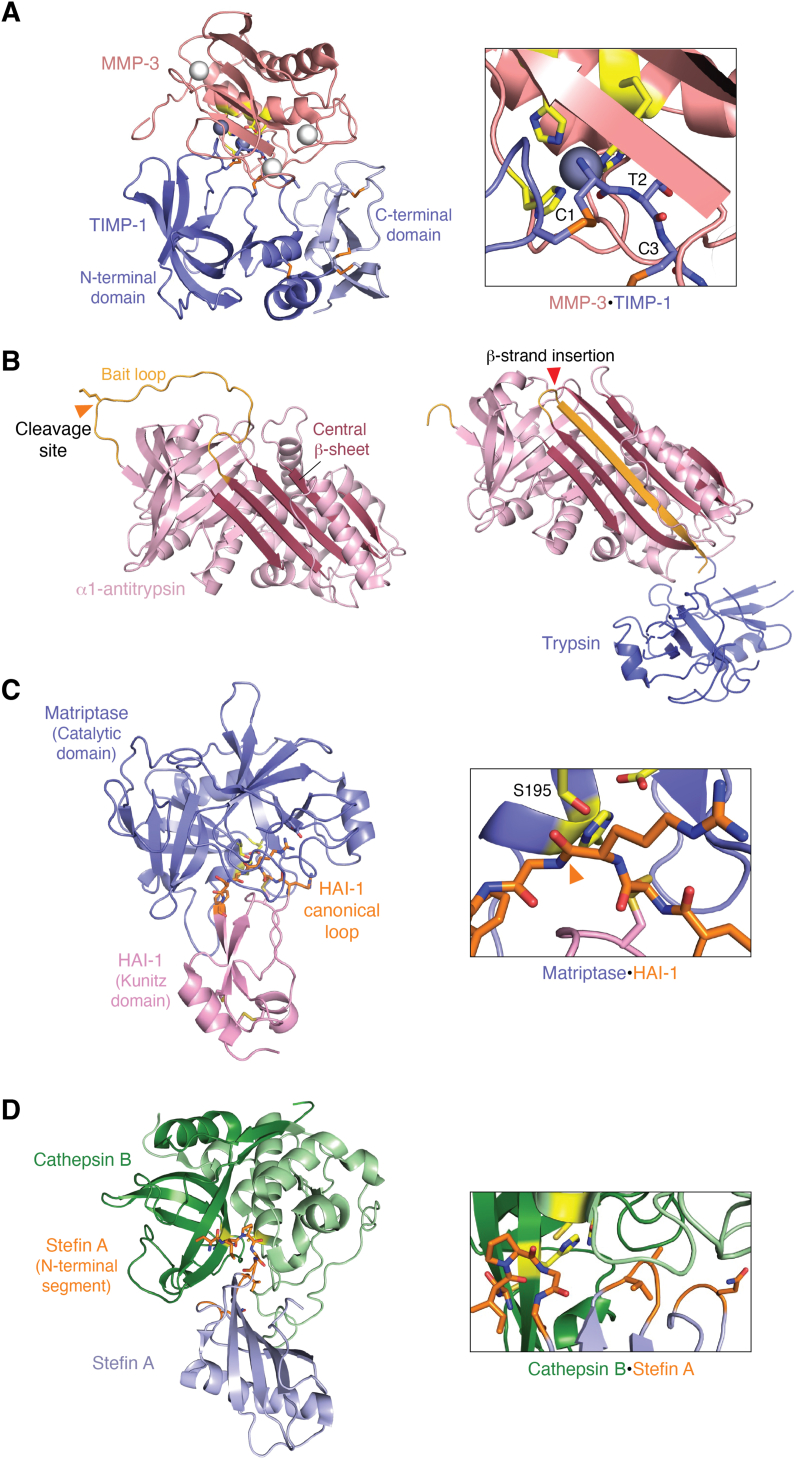
**Protease regulation by endogenous protein inhibitors.***A*, the conserved mode of inhibition of matrix metalloproteinases by TIMPs is exemplified by the structure of the MMP-3 catalytic domain (*salmon;* catalytic residues in *yellow*) bound to TIMP-1 (PDB 1UEA (235)). The TIMP-1 N- and C-terminal domains (*periwinkle* and *pale blue*, respectively) pack side-by-side and interact with the MMP substrate-binding cleft *via* multiple loops (*left panel*). Detail (*right panel*) shows how N-terminal TIMP-1 residues Cys-1, Thr-2, and Cys-3 insert deeply into the catalytic site, with the N-terminal amine coordinating directly to the catalytic zinc ion (*gray sphere*). *B*, structures of α-1 antitrypsin (*light pink*) illustrate the mechanism by which serpins inhibit serine proteases. Native α-1 antitrypsin (*left panel*, PDB 3NE4 (426)) is shown with the bait loop colored in *orange*, and the central β-sheet is shown in *raspberry*. Upon cleavage at the site indicated by the *orange arrow*, the cleaved bait loop becomes inserted into the β-sheet to achieve a more stable protein conformation, as seen in the structure of the trypsin-cleaved α-1 antitrypsin acyl-enzyme (*right panel*, PDB 1EZX (244)). The *red arrow* indicates the position of the new β-strand insertion. The partially disordered trypsin molecule is rendered in *blue*. *C*, exemplary of canonical serine protease inhibitors, the structure of hepatocyte growth factor activator inhibitor-1 (HAI-1) Kunitz domain 1 (*pink/orange*) is shown to inhibit the matriptase catalytic domain (*blue;* PDB 4ISO (427)). The Kunitz domain is a small pear-shaped molecule with the canonical loop protruding at the apex and binding to the protease (*left panel*). Tight binding of the HAI-1 canonical loop (*orange*) in the matriptase active site (*right panel*) positions a reactive site bond (indicated by *orange arrow*) near the catalytic Ser-195, mimicking an ideal substrate. *D*, the inhibitory mechanism of the cystatin superfamily inhibitors is exemplified by the structure of stefin A (*light blue/orange*) bound to cathepsin B (*green*; PDB 3K9M (264)). Stefin A is a wedge-shaped molecule that inserts its N-terminal segment and two hairpin loops deeply into the cathepsin B catalytic cleft (*left panel*). The N-terminal segment fills non-primed substrate-binding subsites to the left of the catalytic site while the hairpin loops occupy primed subsites to the right of the catalytic site (*right panel*, protease-interacting segments shown in *orange* and enzyme catalytic residues shown in *yellow*).

Once activated, proteases are further regulated by endogenous protein protease inhibitors. The balance between proteases and their inhibitors is crucial for maintaining physiological homeostasis. Disruptions in this balance can lead to excessive proteolysis and contribute to cancer progression. The MMPs and adamalysins are regulated by the *tissue inhibitors of metalloproteinases* (*TIMPs*), which in humans consist of a family of four inhibitors with overlapping specificity (234). TIMPs possess two domains that pack side-by-side to form a wedge-shaped molecule, with the thin edge, composed of loops from both domains, binding in the substrate cleft of the protease catalytic domain (Fig. 3*A*, left panel) (235). The core binding epitope at the center of the wedge is formed by the N-terminal C-X-C motif, stabilized by disulfide bonds with two additional cysteine residues within the TIMP molecule. The uncharged N-terminal amine (236) and carbonyl oxygen of Cys-1 coordinate directly with the MMP catalytic zinc ion, displacing the catalytic water molecule and inhibiting enzyme activity (Fig. 3*A*, right panel). Beyond this central interaction, flanking loops from both TIMP domains wrap around the MMP, forming additional favorable molecular contacts with exosites on the catalytic domain that vary among different protease complexes (237).

In addition to their role as protease inhibitors, TIMPs also have protease-independent functions, exhibiting both tumor-promoting and tumor-suppressive effects (234). Accordingly, the relationships between TIMP expression and cancer outcomes can be complex. Nevertheless, regulation of protease activity by TIMPs is critical in controlling many of the tumor-promoting actions of metalloproteases. For example, MMP-9 is often regulated through co-expression with its inhibitor TIMP-1, which also binds to proMMP-9 *via* an exosite and protects the zymogen from activation. However, in the tumor microenvironment, neutrophils can secrete MMP-9 without TIMP-1, leading to its activation and subsequent stimulation of tumor angiogenesis, intravasation, and metastasis (238, 239, 240, 241).

The significance of TIMPs as tumor suppressors is underscored by studies with fibroblasts from quadruple TIMP knockout mice. These fibroblasts, which completely lack TIMP expression, exhibit characteristics of cancer-associated fibroblasts (CAFs), including the ability to augment growth, motility, and metastasis of co-transplanted tumor cells (242). The TIMPless fibroblasts influence tumor cell behavior by secretion of exosomes rich in ADAM-10, while knocking down ADAM-10 in these fibroblasts eliminates their CAF-like properties. Similarly, human CAFs secrete ADAM-10-rich exosomes, and human tumor stroma of multiple tumor types show reduced TIMP levels (242). These findings highlight the critical role of TIMPs in regulating activity of MMPs and adamalysins and how their loss in the tumor microenvironment can promote tumor growth and progression.

S1 family serine proteases are regulated by several types of endogenous protease inhibitors. *Serpins* are large proteins with a fascinating mechanism of inhibition. In their native, inhibitory form, serpins are in a metastable state, presenting a flexible bait loop for the protease to bind and initiate proteolytic cleavage (Fig. 3*B*, left panel) (243). The cleavage of the scissile peptide bond, with formation of an acyl-enzyme intermediate, triggers the serpin to undergo a significant conformational change, incorporating the cleaved bait loop into a large β-sheet, and thereby stabilizing its structure (Fig. 3*B*, right panel) (243, 244). This dramatic conformational change disrupts the protein structure of the attached protease, leading to its permanent inactivation (244). Some examples of serpins that inhibit cancer-associated proteases include the neutrophil elastase inhibitor *alpha-1 antitrypsin*, the uPA inhibitor *plasminogen activator inhibitor-1* (*PAI-1*), and the thrombin inhibitor *antithrombin*.

S1 family members are also regulated by small protein domains known as *canonical serine protease inhibitors*. These inhibitors share a conserved conformation of their protease binding loop that is preconfigured for extremely tight binding to the active site of target proteases (245). The canonical loop binds in the protease active site as an ideal substrate (Fig. 3*C*) and may readily form an acyl-enzyme (246), but is hydrolyzed very slowly and resists dissociation from the protease, leading to re-ligation of the inhibitor peptide bond (247). Crystal structures of protease-bound intact and cleaved inhibitors, supplemented with molecular dynamics simulations, suggest that proteolysis of these canonical inhibitors is dramatically slowed by structural constraints that prevent essential substrate dynamics during proteolysis (248). Examples of canonical inhibitors include *SPINK1*, a trypsin inhibitor (249), and Kunitz protease inhibitor domains found in proteins like *amyloid precursor protein* (*APP*), *amyloid precursor-like protein 2* (*APLP2*), *tissue factor pathway inhibitor-1* and *-2* (*TFPI-1* and *TFPI-2*), all of which regulate proteases of the coagulation cascade (250, 251, 252, 253, 254), and *hepatocyte growth factor activator inhibitor-1* and *-2* (*HAI-1* and *HAI-2*), that regulate the activity of hepatocyte growth factor activator (HGFA) and membrane-anchored serine proteases matriptase, prostasin, hepsin, and TMPRSS2 (255, 256, 257, 258).

Similar to TIMPs, serine protease inhibitors often have pleiotropic functions, including protease-independent signaling activities. Their impact on cancer is complex; for example, PAI-1 and SPINK1 are associated with poor prognosis and may drive cancer progression (259, 260). Importantly, the loss of regulation by endogenous serine protease inhibitors can lead to excess proteolytic activity, promoting cancer. For example, HAI-2 is considered a tumor suppressor, and its loss of expression in cancers including prostate cancer and renal cell carcinoma is linked to tumor progression *via* the dysregulated activity of target proteases (258, 261, 262, 263).

Cysteine cathepsins are also regulated by endogenous protease inhibitors, mainly the *cystatin superfamily* in humans. This family includes intracellular *stefins*, secreted *cystatins*, and cystatin-like domains within *kininogens* (142). The cystatin fold, somewhat similar to TIMPs, has an extended binding epitope formed by the N-terminal segment and two β-hairpin loops, which collectively block the active site cleft of the protease (Fig. 3*D*) (264). The N-terminal segment occupies the nonprimed side, including the hydrophobic S_2_ subsite, while the first loop fills the S_1_ʹ site and the second loop occupies further removed subsites on the primed side. The inhibition of cathepsin B by cystatins involves a two-step mechanism, starting with the formation of an initial encounter complex, followed by conformational changes leading to the stable complex (265, 266). These kinetics can be explained by the structural shifts required of the cathepsin B occluding loop to open the primed side of the cleft for inhibitor binding (264).

Similar to other protein protease inhibitors, cystatins have roles beyond protease inhibition, including both tumor-promoting and tumor-suppressing activities (267). In many tumor types, reduced expression of cystatins or a lower ratio of cystatins to cathepsins is associated with poor prognosis, suggesting a protective role through inhibition of extracellular cathepsin activity (267). Experimental studies support this idea; for example, in the RIP1-Tag2 transgenic mouse model of pancreatic islet cell tumorigenesis, knockout of cystatin C resulted in larger tumors and increased tumor angiogenesis, linked to increased activity of cathepsins and to the cathepsin-mediated degradation of collagen-derived anti-angiogenic peptides (155).

The natural endogenous inhibitors of metalloproteases, serine proteases, and cysteine proteases can themselves serve as substrates for proteases that they are not designed to inhibit. This phenomenon is a significant mechanism through which different proteases exert mutual regulation. This indirect regulation is so widespread that a systems biology analysis of the human protease web identified endogenous protease inhibitors as central to network connectivity (223). Inhibitors often function as critical nodes, enabling different classes of proteases to regulate each other, as further explored in the sub-section, “*Regulation of other enzymes within the protease web – activation of zymogens, degradation of inhibitors*”.

### Regulation *via* localization and complex formation

Control of subcellular location is another critical aspect of protease control. This involves interactions of proteases with proteins, lipids, and carbohydrate structures. Membrane trafficking and protein complex formation help localize extracellular proteases to specific sites, directing their proteolytic activity toward specific substrates. A key example is the concentration of proteases to the cell surface of tumor cells within dynamic actin-based membrane structures known as *invadopodia*. These structures enable tumor cells to efficiently utilize protease activity for the directed degradation of ECM proteins to clear a path for cellular invasion. Proteases are trafficked to invadopodia *via* vesicular transport along microtubules, a process driven by motor proteins and tightly regulated by accessory proteins (268). This process is well-characterized for *MT1-MMP*, a key enzyme in ECM degradation by invadopodia. MT1-MMP and other membrane-type MMPs at invadopodia are indispensable for the ability of invasive breast cancer cells to penetrate the intact, covalently crosslinked basement membrane (23). In the confined environment of 3D crosslinked collagen, when cell migration is restricted by the size and stiffness of the cell nucleus, MT1-MMP is specifically localized to the cancer cell surface in front of the nucleus. Here, invadopodia form to digest confining collagen fibrils (269). These invadopodia can form ring-like structures ahead of the nucleus, encircling the invasive cellular protrusion and expanding over time as MT1-MMP degrades collagen fibers to enlarge the matrix pore (270). Additionally, alternative trafficking patterns in tumor cells can localize MT1-MMP to regions of high plasma membrane blebbing. From these areas, MT1-MMP-rich *tumor-derived microvesicles* are released. The proteolytic activity of these vesicles is also crucial for individual amoeboid cell invasion through dense crosslinked collagen matrices (271).

Pericellular proteolysis employs both proteases with transmembrane anchors, such as membrane-type MMPs, ADAMs, and TTSPs, and a variety of soluble, secreted proteases. These soluble proteases can be localized to the membrane through a variety of binding partners. Soluble proteases of multiple classes (like MMP-2, MMP-9, uPA, cathepsin C) can be trafficked to invadopodia (272, 273, 274) and secreted at targeted degradation sites, or retained on the cell surface by *receptor proteins*. Both soluble and transmembrane proteases interact with specific *integrins*, *tetraspanins*, and other cell-surface receptors that regulate their trafficking and localization at specific points on the cell surface (275). For example, the six members of the *TspanC8* family regulate the trafficking and localization of ADAM-10, influencing its activity toward different specific substrates (276, 277, 278). Similarly, the trafficking, activation, and proteolytic activity of ADAM-17 toward different substrates are influenced by its colocalization and interaction with membrane proteins like *iRhom-1* and *iRhom-2* (279, 280, 281), and several members of the *Tspan8* family (282). Soluble MMP-9 can be localized on the surface of cancer cells by binding to the hyaluronan receptor *CD44* and *α4β1 integrin* (283, 284, 285). uPA is localized by its receptor *uPAR* (273), and procathepsin B is localized by the *annexin II* tetramer on the surface of tumor cells (286).

In some cases, the localization of soluble proteases to the cell membrane may be mediated directly by interactions with lipid bilayer components (287). For example, MMP-7 binds to cell surface *cholesterol sulfate* and partially inserts into the membrane, triggering conformational changes that may lead to allosteric activation, and targeting proteolytic activity toward specific cell-surface proteins and ECM components (288, 289, 290). Furthermore, the trafficking and localization of proteases can be dramatically altered in the cancer setting, as in the case of cathepsin C and cathepsin D, typically lysosomal enzymes, which become redistributed to the cell surface and secreted in many types of cancer cells (233, 291).

*Dimerization and oligomerization* can also regulate protease function. An example of this is seen with MT1-MMP, as discussed earlier in the section “*Maintenance of latency and proteolytic activation*.” MT1-MMP forms dimers that function as receptor/activator complexes with TIMP-2 for activating proMMP-2 (224, 225). Evidence also suggests that MT1-MMP dimerization may enhance its collagenase activity (292). During the invasion of fibrosarcoma cells through 3D collagen matrices, MT1-MMP homodimers are uniquely formed at the leading edge of the invading cells (293), indicating that dimerization possibly regulates cellular invasion. In contrast, ADAM-17 forms homodimers in breast cancer cells, but this dimerization negatively regulates its sheddase activity. The dimeric form is preferentially inhibited by TIMP-3, while signaling pathways that stimulate ADAM-17 sheddase activity, releasing transforming growth factor–α, do so by shifting the monomer-dimer equilibrium toward the active monomeric species (294). MMP-9, which can exist as both monomers and homotrimers (295), also demonstrates how oligomerization affects protease activity. The trimeric form of proMMP-9 has a higher affinity for TIMP-1 than does monomeric proMMP-9, restricting its activation (295). However, trimeric active MMP-9 is less effectively inhibited by alpha2-macroglobulin, a broad-spectrum inhibitor of multiple proteolytic classes (296). Thus, dimerization and oligomerization serve as levers to modulate protease activity *via* multiple mechanisms.

Another mechanism by which proteases in the tumor microenvironment can be localized and brought into contact with their substrates is *via neutrophil extracellular traps* (*NETs*). During inflammation, neutrophils extrude their decondensed chromatin from the nucleus to form these net-like structures. NETs are decorated with neutrophil proteases such as MMP-9, cathepsin G, and neutrophil elastase, and serve to entangle and neutralize pathogens. However, when inflammatory conditions overlap with cancer, NETs can capture and activate circulating dormant tumor cells, thereby promoting cancer metastasis and recurrence (108, 297). The role of proteases attached to NETs is significant in this process. For example, NET-associated MMP-9 and neutrophil elastase have been shown to sequentially cleave laminin-111, exposing a neoepitope that activates tumor cell oncogenic signaling through binding to integrin α3β1 (108). NET DNA acts as a scaffold in this context, preferentially binding to the laminin over other ECM molecules to colocalize this substrate with the proteases at sites of digestion, thereby enhancing their catalytic activity and impact on tumor progression.

## Substrates in the tumor microenvironment—extracellular protease actions at the scene of the crime

In the tumor microenvironment, proteases play a pivotal role at the molecular level through the recognition and cleavage of specific protein substrates (key proteases and substrates summarized in Table 1). The extracellular space around a developing tumor exposes proteases to a diverse array of potential substrates. These include protein components of the ECM, soluble proteins secreted by tumor and stromal cells, and extracellular domains of transmembrane and membrane-associated proteins on the plasma membranes of these cells. Extracellular proteases significantly influence many aspects of tumor growth, progression, and metastasis by proteolyzing of numerous proteins within these substrate categories. Some of these actions leverage the broad degradative capabilities of proteases to dismantle barriers and forge pathways for the invasion of tumor cells. However, many critical tumor-promoting activities of extracellular proteases involve highly specific and limited proteolysis. This precision-targeted activity focuses on particular recognition and cleavage sites to activate latent signaling molecules or to directly initiate signaling pathways through proteolysis.

### Degradation and remodeling of the extracellular matrix

The extracellular matrix (ECM), composed of around 300 unique proteins, proteoglycans, and glycoproteins, is organized into two primary forms: the porous *interstitial matrix*, which connects cells in the stroma, and the dense, sheet-like *basement membrane*, which underlies epithelial and endothelial cell sheets and delineates tissue compartments (298). Tumor cells, immune cells, fibroblasts, and other cells in the tumor microenvironment produce extracellular proteases that reshape and remodel the ECM, facilitating tumor initiation, growth, and progression through the cleavage of a multitude of ECM proteins and glycoproteins (298).

One of the best-appreciated proteolytic functions in cancer is the *degradation of the basement membrane*, enabling tumor cells to invade into stromal tissue, a key phenomenon that marks the progression to malignancy. Basement membranes primarily consist of covalently crosslinked nonfibrillar *collagen IV* intertwined with a network of polymeric *laminin* (298). The disruption of this barrier initially involves specialized collagenases, notably membrane-associated MMPs MT1-MMP, MT2-MMP, and MT3-MMP (23, 299, 300). Additional proteases, including uPA, plasmin, and the gelatinases MMP-2 and MMP-9, further degrade the basement membrane by cleaving collagen fragments and other matrix components, and/or by activating other protease zymogens (25, 26). Cysteine cathepsins also play a role, particularly during angiogenesis. For instance, vascular endothelial growth factor (VEGF) upregulates cathepsins B, S, and L in pericytes, leading to the degradation of the vascular basement membrane of precursor venules, which then expand into larger vessels in a mouse model simulating the earliest stages of tumor angiogenesis (148).

Once tumor cells invade the stroma, passage through the dense interstitial matrix requires cleavage of *fibrillar collagens types I, II, and III*. This process requires the action of collagenases, which include MMP-1, MMP-8, MMP-13, MT1-MMP, MT3-MMP, and cathepsin K (301). While these collagenases exhibit some functional redundancy (24), they can produce distinct cleaved fibril morphologies and differentially cleave other ECM substrates, leading to unique cellular interactions with ECM and diverse functional outcomes (302). Importantly, experiments with fibroblasts and tumor cells have shown that the specific involvement of MT1-MMP in collagenolysis is essential for cellular invasion through 3D fibrillar collagen matrices (22, 24). This critical role in linking collagen degradation with directional motility is made possible by the regulated trafficking and spatial localization of MT1-MMP to dynamic actin-based invadopodia on the leading edge of invasive cells, as previously detailed in the section “*Regulation via localization and complex formation*”.

Beyond collagen, the degradation of other protein components of ECM is also crucial for tumor growth, progression, and metastasis. For example, the degradation of *laminin-111* by MMP-9 disrupts the apicobasal polarity of breast cells, fostering invasive growth in 3D cultures (303). Furthermore, mammary tumor xenografts with reduced MMP-9 expression exhibited higher laminin-111 levels, coinciding with suppression of tumor growth; this finding highlights the importance of laminin-111 as a functional MMP-9 substrate *in vivo* (303). Another key basement membrane component, *Laminin-332*, which anchors epithelial cells *via* integrins at hemidesmosomes, can be cleaved by the S1 family TTSP hepsin (304, 305). This cleavage disrupts apicobasal polarity in mammary organoids and accelerates tumor formation in a myc-dependent mammary tumorigenesis model, pointing to the relevance of laminin-332 degradation in tumorigenesis (305). In prostate cancer models, the TTSP TMPRSS2 degrades *nidogen-1*, a basement membrane glycoprotein that cross-links collagen IV and laminin. This degradation facilitates prostate cancer cell invasion and metastasis (306).

*Thrombospondin-1*, with anti-angiogenic and anti-metastatic properties, is another critical ECM substrate (307, 308). In lung inflammation models, neutrophil-secreted serine proteases neutrophil elastase and cathepsin G degrade thrombospondin in the mouse lung microenvironment, leading to increased lung metastasis of melanoma (107). Mice lacking neutrophil elastase and cathepsin G in bone marrow cells showed preservation of thrombospondin-1 and reduced metastasis, emphasizing these proteases’ role in preparing the lung metastatic niche.

Protease action on ECM proteins can also involve *limited proteolysis* to reveal *cryptic epitopes* that mediate new functions. Proteases capable of cleaving *laminin-332*, including MMP-2, MT1-MMP, and TTSPs hepsin and matriptase, generate fragments that enhance cancer cell migration (304, 309, 310, 311). Neutrophil elastase and MMP-9, both associated with NETs in lung inflammation, sequentially cleave *laminin-111* to expose a cryptic epitope recognized by α3β1 integrin on dormant breast cancer cells (108). This integrin activation triggers oncogenic signaling, awakening the cancer cells from dormancy and promoting metastatic lesion growth. An antibody targeting this laminin-111 cryptic epitope effectively blocked NET-induced β1 integrin activation, inhibiting the reactivation of dormant breast cancer cells both *in vitro* and *in vivo* (108).

Another cryptic epitope is crucial in the aggressive invasion of gliomas. The central nervous system possesses a unique ECM composed of hyaluronic acid and associated glycoproteins and proteoglycans including *brevican*, a neural-specific proteoglycan that is highly upregulated in gliomas. M12 family proteases ADAMTS-4 and ADAMTS-5 cleave brevican to expose a cryptic epitope that promotes glioma cell adhesion, motility, and invasion into the brain, in part through binding to tumor cell surface fibronectin (42, 43, 44).

Limited proteolysis of ECM proteins can also generate soluble bioactive fragments, known as *matrikines*, with novel functions. One example is the processing of *laminin-332* by MMPs, which not only facilitates cellular invasion but also releases a soluble domain from the laminin-332 γ2 chain. This domain, containing several EGF-like repeats, has been found to interact with EGFR, activating signaling pathways that enhance cell motility (312). Additionally, cathepsin S cleaves laminin-332 in a mouse model of pancreatic tumorigenesis to produce pro-angiogenic fragments of the γ2 chain (155). This model also demonstrated the ability of cathepsin S to cleave and deactivate several anti-angiogenic matrikines derived from collagen IV. Cathepsin S knockout impaired tumor angiogenesis in this model, demonstrating the importance of this protease in stimulating tumor progression through the regulation of matrikines (155).

While intact *thrombospondin-1* exhibits anti-angiogenic and anti-metastatic properties, its proteolytic processing releases a variety of matrikines with divergent activities (313). Many proteases can release thrombospondin fragments containing an N-terminal heparin-binding domain (313), which are powerful stimulators of angiogenesis (314, 315). MT1-MMP production of thrombospondin-1 matrikines is crucial for intussusceptive angiogenesis, a microvasculature expansion observed in development, inflammatory diseases, and certain cancers (316). In a mouse model of inflammatory colitis, endothelial cell-derived MT1-MMP cleaved thrombospondin-1, releasing a 50 kDa N-terminal fragment and a larger C-terminal fragment. These fragments signal through CD47 and αvβ3 integrin, promoting nitric oxide production and pathological intussusceptive remodeling (316). These examples highlight how proteolytically derived fragments from ECM molecules can signal and influence cancer progression.

### Release, activation, and inactivation of soluble signaling molecules

The ECM serves as a reservoir for many soluble growth factors and signaling molecules, which remain sequestered when bound to matrix proteins, glycoproteins, and proteoglycans. Their release often depends upon the action of proteases and/or glycosidases. A key example is the regulation of *transforming growth factor-β* (*TGFβ*), a multifunctional cytokine. While TGFβ suppresses the growth of early neoplastic lesions, it also contributes to metastasis, angiogenesis, and suppression of anti-cancer immune responses in later cancer stages. Latent TGFβ, largely sequestered within the ECM, is tightly bound to a *latency-associated peptide* (*LAP*) that is linked *via* disulfide bonds to a *latent TGFβ binding protein* (*LTBP*). LTBPs attach to matrix components like fibronectin, fibrillin-1, and heparan sulfate, and can become crosslinked into the ECM (317). TGFβ activation involves its dissociation from LAP, which is initiated by integrin binding of the latent complex (317). A number of extracellular proteases, including plasmin, neutrophil elastase, mast cell chymase, MMP-2, MMP-9, and MT1-MMP, can enhance TGFβ activation and bioavailability in different tissues by cleaving LAP, LTBPs, or other matrix proteins (318). For example, MMP-9, when bound to CD44 on the cell surface of mouse mammary carcinoma cells, promotes cellular invasion and angiogenesis by activating latent TGFβ, likely through direct cleavage of LAP (319). Activated osteoclasts, which play a role in bone destruction during bone metastasis, can mobilize and activate TGFβ from bone ECM by cleaving *LTBP-1* with MMPs and serine proteases (320). Similarly, activated endothelial cells release latent TGF-β complexes from the subendothelial matrix by cleaving LTBP-1, with MT1-MMP identified as the responsible protease (321). Additionally, the TTSP hepsin, frequently overexpressed on breast and prostate cancer cells, stimulates TGFβ signaling by degrading *fibronectin*, thus freeing latent TGFβ from the ECM (322).

*VEGF*, a major regulator of pathological angiogenesis in cancer, is regulated *via* sequestration in the ECM, binding to heparan sulfate proteoglycans. MMPs or plasmin can release active VEGF fragments, enabling them to bind to VEGF receptors on endothelial cells and stimulate angiogenesis (323, 324). MMP-9 proteolysis is critical for VEGF mobilization in several models of tumorigenesis. For example, MMP-9 secreted by infiltrating neutrophils triggers the angiogenic switch in a pancreatic carcinogenesis model (238, 325), while in cervical carcinogenesis, MMP-9 from macrophages plays a similar role (326). *Fibroblast growth factor 2* (*FGF-2*), also sequestered in the ECM by heparan sulfate proteoglycans, influences tumor growth and angiogenesis (327, 328). In cell culture models, FGF-2 can be proteolytically released by plasminogen activators and plasmin, stimulating endothelial cell growth (327); breast cancer cells can also release FGF-2 *via* cathepsin D (184). Moreover, thrombin can cleave high molecular weight isoforms of FGF-2 to create a lower molecular weight variant with enhanced ability to stimulate endothelial cell migration and proliferation (329). These instances are just a few examples of the many growth factors within the ECM, whose bioavailability is modulated by extracellular proteases in the tumor microenvironment.

Extracellular proteases also regulate the activation and inactivation of many soluble signaling molecules, significantly impacting cancer growth, progression, and interaction with the immune system. *Hepatocyte growth factor* (*HGF*) and *macrophage-stimulating protein* (*MSP*) are examples of growth factors activated by proteases. These secreted proteins are initially produced as inactive precursors and require cleavage to generate the active signaling heterodimer, where the α and β chains remain connected *via* a disulfide bond (330). Both HGF and MSP are non-proteolytic homologs within the S1 serine protease family that lack catalytic activity due to mutations of catalytic residues. Their activation as signaling proteins involves cleavage at the same site responsible for zymogen activation in other S1 family proteases. This cleavage induces similar conformational changes and structure stabilization in the pseudoprotease domain (330), as described above for active S1 proteases in the section “*Maintenance of latency and proteolytic activation*”. The conformational change enables activated HGF or MSP to bind to and promote dimerization of their respective receptor tyrosine kinases, MET and RON, initiating cell signaling that can drive malignant transformation and cancer progression (331, 332). The TTSP matriptase is a critical activator of HGF, shown to promote c-MET mediated carcinogenesis and cancer progression in mouse models of breast cancer and skin squamous cell carcinoma (97, 98, 99, 100, 102). Similarly, the TTSP TMPRSS2, highly expressed in metastatic prostate cancers, efficiently activates HGF, contributing to prostate cancer metastasis through c-MET signaling in a mouse model (101). TTSPs matriptase and hepsin have also been implicated as pathologically relevant activators of MSP, mediating signaling through RON (333, 334).

*Platelet-derived growth factors* (*PDGFs*) are another family of growth factors pivotal in cancer development and progression. *PDGF-C* and *PDGF-D* are secreted as latent homodimers, requiring proteolytic removal of an N-terminal domain to activate the growth factor domain dimers that signal through α-PDGFR and β-PDGFR, respectively (335, 336). Tissue plasminogen activator (tPA) has been identified as the primary activator of PDGF-C in fibroblasts (337), while tPA, uPA, and matriptase contribute to PDGF-C activation in ER-positive breast cancer cells (338). Recent mouse model studies of ER-positive breast cancer demonstrate that low levels of PDGF-C signaling in the lung microenvironment of young mice support the survival of dormant disseminated tumor cells. In contrast, increased PDGF-C and its activator tPA in the lungs of aged mice promote metastatic outgrowth of these tumor cells (339). A similar age-related upregulation of PDGF-C and tPA is observed in human lung tissues, potentially explaining the extended latency of dormant disseminated ER+ breast cancer cells and the significant risk of late metastatic recurrence (339). PDGF-D signaling is linked to prostate cancer progression and recurrence, with uPA and matriptase identified as the key activating proteases (340, 341).

Many *chemokines* undergo proteolysis, where N-terminal truncation can activate or inactivate them depending on the chemokine and cleavage site, leading to complex biological outcomes in cancer. For example, neutrophil attractant CXC chemokine *interleukin-8* (*IL-8*) is activated through processing by MMP-9, plasmin, or thrombin. In a positive feedback loop, neutrophil-produced MMP-9 removes the N-terminal 6 residues from IL-8, increasing its affinity for the receptor CXCR1 and its potency for neutrophil activation by 10- to 27-fold (342). Proteolytic activation of IL-8 is of importance in the tumor microenvironment, since IL-8 is secreted by many tumor cells and signals through CXCR1 on cancer cells, endothelial cells, neutrophils and tumor-associated macrophages, stimulating multiple aspects of cancer progression (343). Conversely, an example of inactivating cleavage involves the processing of *CXCL10* and *CXCL11* by DPP IV, which removes their N-terminal dipeptide, converting these CXCR3 agonists into antagonists and reducing their chemotactic potency by more than 10-fold (114). CXCL10 is crucial in tumor immunity and immunotherapy responses, while its inactivation by DPP IV creates a tumor-permissive environment; inhibiting DPP IV has been shown to improve tumor immunity and immunotherapy in mouse models of hepatocellular carcinoma and melanoma (115, 116).

Several signaling molecules are initially expressed in latent form as membrane proteins on the cell surface, requiring proteolysis for the release of the soluble, active ligands. Prominent examples include the members of the *epidermal growth factor* (*EGF*) *family*, type I membrane proteins that upon cleavage by cell surface proteases release an extracellular ectodomain that functions as a soluble growth factor and ligand for the *EGF receptor (EGFR)* family of receptor tyrosine kinases (344). Some of the EGF family members for which ectodomain shedding mediates protumorigenic activities in cancer include *EGF*, *transforming growth factor alpha* (*TGF-α*), *heparin-binding EGF-like growth factor* (*HB-EGF*), and *amphiregulin*. The primary sheddases responsible for release of these growth factors are members of the ADAM family, in which ADAM-10 is the major sheddase of EGF, while ADAM-17 is the major sheddase of TGF-α, HB-EGF, and amphiregulin (36). ADAM-17 cleavage of TGF-α is crucial for activation of EGFR by this growth factor and is essential for tumor development in a mouse model of TGF-α-dependent tumorigenesis (35).

EGF-like growth factors shed from the surface of tumor cells also mediate paracrine signaling to stromal cells, promoting tumor growth. A recent study investigating how tumor cells influence tumor-associated macrophages, leading to their acquisition of protumorigenic characteristics, revealed that this process is driven by HB-EGF and amphiregulin shedding from tumor cells through ADAM-17. These growth factors then signal through EGFR on macrophages (345). Other proteases are also involved in EGF-like ligand shedding in different cancer-relevant settings. For example, MMP-7 can release HB-EGF from murine mammary epithelial cells, triggering autocrine signaling through the ErbB4 receptor and leading to malignant transformation (346). Additionally, studies using a breast cancer to bone metastasis model implicated MMP-1 and ADAMTS-1, secreted by breast cancer cells, as the proteases responsible for shedding TGF-α, HB-EGF, and amphiregulin. These ligands then activate EGFR signaling in bone stromal cells, facilitating bone metastasis (45).

*Receptor activator of nuclear factor-kappa B ligand* (*RANKL*) is another membrane protein that, when cleaved, releases a soluble ectodomain mediating signaling activities. In bone, osteoblasts produce RANKL, which then signals through its receptor RANK on osteoclasts and their precursors to promote osteoclast differentiation and activation. In bone stromal cells and primary osteoblasts, ADAM-10 or MT1-MMP can shed RANKL, releasing the soluble ectodomain (38). In the context of cancer, the interaction between tumor cells and bone stroma disrupts bone homeostatic processes. For instance, metastatic prostate cancer cells induce osteoblasts to upregulate RANKL, activating osteoclasts. These activated osteoclasts then secrete MMP-7 at the tumor-bone interface. In a positive feedback loop, MMP-7 has been shown to shed soluble active RANKL, further driving osteoclast activation and osteolysis at the tumor-bone interface in a rat model (347). A similar phenomenon is observed in breast cancer bone metastasis mouse models, where cathepsin G secreted by osteoclasts acts as a sheddase of RANKL, contributing to further osteoclast activation and mammary tumor-induced osteolysis (110). Tumor cells can also express the RANKL/RANK system, and shedding of soluble RANKL can mediate oncogenic signaling pathways. For example, RANKL shed from prostate cancer cells by MT1-MMP was shown to initiate an autocrine signaling loop leading to enhanced tumor cell migration (348). These examples highlight the multifaceted role of RANKL shedding in cancer progression, particularly in the context of bone metastasis.

### Cleavage of transmembrane proteins

Protease cleavage of cell membrane proteins contributes to tumorigenesis and tumor progression beyond merely shedding soluble signaling molecules. *Protease-activated receptors* (*PARs*) are a critical group of protease substrates in cancer. This family of 4 G protein-coupled receptors is activated by proteolysis of their extracellular N-terminal domain. Cleavage at a specific site within this domain exposes a cryptic epitope at the new N-terminus, which acts as a tethered ligand that docks with a binding site comprised of extracellular loops of the receptor, triggering transmembrane signaling (65, 349). PAR1, PAR3, and PAR4 are typically activated by thrombin, although various other proteases can cleave and activate the PARs in different physiological and pathological settings, sometimes targeting noncanonical cleavage sites, leading to biased agonism with distinct signaling consequences (349).

*PAR1* is widely upregulated in human cancers, and protease signaling through PAR1 contributes to tumorigenesis, cancer growth, motility, invasion, metastasis, and angiogenesis. Early investigations found that melanoma cells could activate thrombin, which signaled through PAR1 to stimulate proliferation and enhance experimental metastasis (63); this mechanism and its impact on metastasis have been corroborated in multiple other tumor cell types (64). More recent studies using a genetically engineered mouse model of pancreatic ductal adenocarcinoma showed that thrombin-PAR1 signaling in tumor cells mediates immune evasion mechanisms and fosters an immunosuppressive tumor microenvironment (60, 350). In mouse models of colon adenocarcinoma, thrombin-dependent tumor growth was reduced when PAR1-expressing tumor cells were implanted into PAR1-deficient mice, revealing that thrombin-PAR1 signaling in stromal cells also contributes to tumor growth (59). In mouse models of osteolytic breast cancer metastasis to bone, PAR1 signaling in monocyte osteoclast precursors was found to drive chemotaxis, increasing osteoclast precursors and mature osteoclasts at the tumor-bone interface (111), with cathepsin G, upregulated at the tumor-bone interface in both tumor cells and monocytes, mediating this process (111). Additionally, PAR1 activation is not limited to serine proteases; MMP-1 can activate PAR1 *via* cleavage at a noncanonical site, initiating a distinct downstream signaling profile (351). In mouse models of breast cancer, MMP-1 derived from stromal fibroblasts was shown to activate tumor cell PAR1, promoting tumor growth and invasion (70).

*PAR2* is also frequently upregulated in human cancers, playing a significant role in inflammatory signaling and contributing to tumorigenesis, cancer growth, motility, invasion, metastasis, and angiogenesis (349, 352). Typically activated by trypsin, PAR2 can also be activated by other proteases in different biological contexts. Tumor-associated trypsin, highly expressed by colon, pancreatic, and ovarian cancer cells, activates PAR2 signaling, stimulating mitogenic pathways and cell proliferation (75, 76, 353, 354). The trypsin isoform PRSS3/mesotrypsin is also implicated in PAR2 signaling, leading to tumor cell proliferation and survival in esophageal adenocarcinoma (81). Beyond trypsins, the TTSP matriptase is a significant direct mediator of PAR2 signaling, cleaving at the canonical site (103, 352, 355). In skin squamous cell carcinogenesis models, matriptase signaling through PAR2 on keratinocyte stem cells induces pro-inflammatory and pro-tumorigenic effects essential for the progression of pre-malignant cells to malignancy (106). In normal skin, PAR2 colocalizes with matriptase, which is largely found in complexes with inhibitor HAI-1. However, in squamous cell carcinoma, HAI-1 downregulation leads to active matriptase, potentially enhancing PAR2 signaling (356). The matriptase/HAI-1 ratio is also important in ovarian cancer, where low HAI-1 and high matriptase correlate with metastasis. Recent studies in ovarian cancer models show that matriptase-activated PAR2 signaling disrupts cell-cell adhesion, aiding the dissemination of ovarian cancer spheroids and increasing metastasis (357). Intriguingly, the role of matriptase in PAR2 signaling extends beyond autocrine stimulation of tumor cells. When deregulated by HAI-1 silencing, soluble active matriptase shed from oral squamous cell carcinoma cells can transactivate fibroblast PAR2, enhancing fibroblast migration (358). The link between cancer-associated fibroblast expression of PAR2, invasive tumor histology, and poor patient outcomes suggests that this soluble matriptase-PAR2 paracrine signaling axis contributes to oral squamous cell carcinoma progression (358).

Other proteases, such as KLKs, are also involved in PAR signaling in cancer. In prostate cancer, KLKs like KLK2 and KLK4, highly expressed by cancer cells, signal through PAR2, and KLK4 additionally signals through PAR1, stimulating proliferation (93, 94). KLK4 secreted by prostate cancer cells can signal through PAR1 expressed on stromal fibroblasts, inducing cytokine release from activated fibroblasts, which in turn stimulates mitogenic signaling in cancer cells, driving tumor growth (95).

Protease cleavage can also modulate the activities of other types of *membrane receptors*. For example, *EphA2*, a receptor tyrosine kinase that binds to membrane-bound ephrin ligands on adjacent cells, regulates several processes essential for the maintenance of epithelial architecture. In breast and ovarian cancer cells, EphA2 is overexpressed and constitutively cleaved by MT1-MMP on the cell surface, releasing an N-terminal fragment (359, 360). This proteolysis of EphA2 by MT1-MMP disrupts its capacity for ligand-dependent signaling and instead promotes ligand-independent signaling. This transformation converts EphA2 from a tumor suppressor into an oncoprotein, enhancing malignant growth, cell migration and single-cell invasion (359, 360).

*CUB domain-containing protein 1* (*CDCP1*) is another transmembrane receptor overexpressed in many carcinomas. CDCP1 initiates oncogenic signaling upon phosphorylation of its intracellular domain and complex formation with kinases Src and PKCδ. However, these processes only begin after the extracellular domain is cleaved by serine proteases at a specific pair of sites (361, 362, 363). Protease cleavage of CDCP1 enhances cell survival, migration, dissemination, and metastatic colonization in prostate and breast cancer models (364, 365, 366). Plasmin and uPA are primarily implicated as the activators of CDCP1 signaling *in vivo*. In an experimental metastasis model, wild-type mice exhibited CDCP1 cleavage in lung tissue, leading to signaling pathway activation and robust colonization by human cells. Conversely, plasminogen-knockout mice showed intact CDCP1, with abrogation of signaling and greatly reduced lung colonization. These effects were reversed by administration of physiological levels of purified plasmin (364). A recent study employed activity-based probes mimicking the CDCP1 cleavage site to trap candidate proteases in pancreatic cancer cell cultures, identifying tumor cell-produced uPA and bovine plasmin from the culture medium (367). Furthermore, knocking down uPA in pancreatic, prostate, and ovarian cancer cell lines prevented CDCP1 cleavage, suggesting that uPA is a major *in vivo* regulator of CDCP1, both through direct cleavage and by activating plasminogen to plasmin (367).

*Adhesion proteins* are another category of cell membrane proteins targeted by proteases to mediate tumor promoting effects. One well-documented example is *E-cadherin*, which mediates cell-cell adhesion at adherens junctions of epithelial cells through homodimerization between neighboring cells. These contacts are critical for maintaining epithelial architecture, and proteolytic targeting of the extracellular component of E-cadherin can trigger epithelial-mesenchymal transition and promote cell migration and invasion (29, 30). The released extracellular fragment of E-cadherin also contributes to these protumorigenic effects, both through interference with the cell-cell junctions of other cells, and *via* signaling through ErbB receptors (30, 32, 368). Extracellular proteases implicated in E-cadherin cleavage and associated tumorigenic processes include ADAM10, ADAM15, MMP-3, MMP-7, MMP-9, and the cysteine cathepsins B, L, and S (29, 30, 31, 32, 37, 152, 357, 368). KLKs 6 and 7 have also been implicated, although for KLK6 the shedding of E-cadherin appears to be indirect, *via* activation of ADAM10 (89, 369).

Other members of the cadherin superfamily have been implicated as protease substrates in cancer, including *desmoglein-1* and *-2*, which mediate cell-cell adhesion at desmosomes of epithelial cells. Ectodomain shedding of desmoglein-1 by KLK5 and desmoglein-2 by ADAM-17 contributes to loss of cell cohesion in oral squamous cell carcinoma models (39, 90). Matriptase was found to cleave desmoglein-2 in colon cancer cells (370), and KLK7 cleaves desmoglein-2 in pancreatic cancer cells (91). *Junctional adhesion molecules* (*JAMs*), members of the immunoglobulin superfamily, also represent proteins involved in cell-cell adhesion that are potential targets of extracellular proteases. *JAM-B*, highly expressed in human brain microvascular endothelial cells, is essential for cell-cell adhesion at tight junctions to maintain barrier function. Cathepsin S specifically mediates metastatic breast cancer cell transmigration across the blood–brain barrier in brain metastasis models by cleaving the JAM-B extracellular domain, disrupting tight junction integrity (153).

### Regulation of other enzymes within the protease web—activation of zymogens, degradation of inhibitors

The intricate network of proteases and their proteinaceous inhibitors, spanning multiple classes and families, form a complex “protease web,” where proteases often regulate each other’s activity through activating or inactivating cleavage events (223). Proteolytic activation of protease zymogens is a common theme, as highlighted earlier in the section, “*Maintenance of latency and proteolytic activation*”. Classic examples include the protease activation cascades leading to thrombin activation and the activation of plasmin by plasminogen activators tPA and uPA. In cancer, the upregulation of diverse proteases, along with changes in their trafficking and localization, can perturb the protease web resulting in activation of protease zymogens by nonphysiological activators. Activation cascades in the tumor microenvironment often involve proteases from different classes and families. For example, a cascade featuring sequential activation of uPA, plasmin, MMP-3, and MMP-9 enhances breast carcinoma cell invasion (371). Similarly, cascades involving uPA or tPA activation of plasmin, which in turn activates MMPs, have likewise been implicated in collagen degradation and basement membrane invasion by many types of cancer cells (25, 26, 372).

Upstream mediators of uPA activation include the TTSPs matriptase and hepsin in ovarian and prostate cancer models, respectively (104, 105), and cell surface-associated cathepsin B in ovarian cancer cells (373). Matriptase and hepsin can autoactivate on the cell surface (213), and active matriptase can be further increased *via* activation by TMPRSS2 in prostate cancer cells (306). Cysteine cathepsin B secreted by colon cancer cells is activated by aspartic cathepsin D (185), while cathepsin D itself is activated by cathepsins B and L (187). Distinct protease activation cascades have been implicated in different metastatic niches; for example, the prometastatic activities of MMP-9 at the tumor-bone interface in metastatic breast cancer osteolytic lesions depend on proMMP-9 activation by cathepsin G from osteoclasts (109). Additionally, the proteolytic activation of enzymes in the tumor microenvironment extends beyond proteases. For example, heparanase, a glycoside hydrolase that promotes invasion and metastasis by cleaving heparan sulfate, is activated by cathepsin L secreted by tumor cells and fibroblasts (154).

The protease web encompasses not only proteases but also their endogenous proteinaceous inhibitors. These inhibitors can be substrates for other proteases that are not susceptible to inhibition (223). This inactivating cleavage of inhibitors by a non-targeted protease creates a pathway for indirectly upregulating the activity of one or multiple other proteases. For instance, TIMP-1 forms a complex with its physiological target proMMP-9 to limit activation; however, TIMP-1 within this complex can be preferentially inactivated upon cleavage by neutrophil elastase, rendering proMMP-9 vulnerable to activation by MMP-3 (374). The inactivating cleavage of TIMP-1 and TIMP-2 by cathepsin B can also contribute to enhanced MMP activity and increased MMP-dependent angiogenesis (375).

The cystatins are another family of inhibitors regulated by proteolysis. A proteomics approach for the discovery of MMP-2 substrates in the fibroblast secretome revealed multiple protease inhibitors among the novel substrates, including cystatin C. Notably, cystatin C cleavage by MMP-2 reduced its inhibitory potency towards cysteine cathepsin targets (376). Cystatin C is similarly cleaved by aspartic cathepsin D secreted by breast cancer cells, resulting in greatly increased activity of cathepsin B in the breast cancer cell secretome (186).

PRSS3/mesotrypsin is an isoform of trypsin that is not just resistant to inhibition by canonical serine protease inhibitors but can cleave these inhibitors, greatly diminishing their potency toward other target proteases (77, 82, 83, 377, 378). Protease inhibitors that are substrates of mesotrypsin include APPI, APLP2, TFPI-1, and TFPI-2, inhibitors of coagulation pathway serine proteases, along with HAI-2, an inhibitor of matriptase, hepsin, prostasin, and HGFA, and bikunin, an inhibitor of cell surface-bound plasmin (77, 82, 83). Mesotrypsin can thus act as a key node in the protease web, regulating through protease inhibitor cleavage the activity of a spectrum of serine proteases. These proteases in turn mediate growth factor activation, PAR signaling, and ECM degradation, contributing to mesotrypsin’s roles in tumor growth, progression, and metastasis (79, 80).

## Identifying the villains and their modus operandi—an evolving forensic toolbox

To understand the role of a protease in cancer, it is crucial to identify the substrates it acts upon to produce pathogenic effects. No single research approach can definitively ascertain the specific substrates and cleavage events mediated by individual proteases while simultaneously demonstrating the biological and pathological significance of these events. Genetic knockout and overexpression mouse models have been critical for demonstrating the importance of some individual proteases in tumorigenesis and tumor progression. However, the complexity of the protease web *in vivo* often makes it difficult to attribute specific protein cleavages in these models to a single protease. *In vitro* studies using purified enzymes and protein substrates can reveal specific cleavage sites and kinetics, but cannot confirm whether these cleavage events occur *in vivo* or are significant drivers of disease. The most robust evidence implicating specific proteases, substrates, and their pathological mechanisms in cancer combines insights from multiple disciplines and approaches. Such a strategy might include identifying substrates through unbiased proteomics approaches, validating their relevance in cancer using *in vivo* models and analyses of patient tumors, and establishing direct cleavage relationships between enzymes and substrates *via in vitro* biochemical assays with purified proteins. The multitude of extracellular proteases involved in the protease web of cancer poses a significant challenge to clearly attributing specific cleavage events and understanding their mechanistic significance. However, evolving technologies in the areas of proteomics, probe development, and chemical biology are increasingly capable of addressing these challenges, helping to unravel protease-substrate relationships and accelerating progress in this field.

### Substrate profiling and identification

One of the most effective and unbiased approaches for discovering protease substrates in biological systems comes from mass spectrometry-based *degradomics* (379). This specialized branch of proteomics seeks to identify and/or quantify proteolytic cleavage events across the proteome. Degradomics for substrate discovery typically involves comparing proteomes treated with a specific protease to untreated ones, or contrasting proteomes from models overexpressing or deficient in the protease with control models exhibiting native protease expression. The goal is to identify candidate substrates and cleavage sites that are differentially processed depending on the expression or activity of the protease. To increase the sensitivity of detecting peptides resulting from relevant cleavage events, several strategies focus on enriching the proteome sample for neo-termini created by these events (2, 379, 380). Common approaches involve chemically blocking all primary amines in the proteome samples, including both native and processed N-termini. This step incorporates an isotopic label or isobaric proteomics tag, enabling differential labeling of multiple samples for comparison. The samples then undergo digestion by trypsin or another digestive protease to produce peptides suitable for LC-MS analysis. Next, peptides with unlabeled N-termini are depleted, enriching for the labeled N-termini. Subsequent LC-MS analysis and assignment using specialized bioinformatics tools and databases enables the identification of the parent proteins showing differential cleavage between samples, indicating potential substrates, along with the specific sites of cleavage (2, 379, 380).

Three of the most common of these *N-terminomics* approaches, differing in their modification and enrichment chemistries, are *terminal amine isotopic labeling of substrates* (*TAILS*) (381), *combined fractional diagonal chromatography* (*COFRADIC*) (382), and enzymatic N-terminal labeling employing an engineered peptide ligase (383). These approaches have shed light on the substrate ensembles of a select number of extracellular proteases implicated in cancer progression. One of the most comprehensive studies reported to date employed TAILS with 8-plex iTRAQ labeling to profile the neo-N-termini in tumors from the Rip1-Tag2 pancreatic cancer model. This study compared tumors from mice with six different cysteine cathepsin knockout genotypes (149). The findings indicated that for these enzymes, overall degradation was more prevalent than limited proteolytic processing. A smaller group of putative substrates was identified as stable proteolytic products, which varied among the different cathepsin knockouts, indicating distinct, nonredundant functions of these enzymes.

Chemical biology offers innovative tools to enhance substrate identification, which is particularly useful for cell surface proteins which, despite their critical roles in cancer, are frequently underrepresented in mass spectrometry-based degradomics due to low abundance. To address this issue, *cell surface N-terminomics* introduces labeling and enrichment techniques specifically for the extracellular cell surface proteome. The approach employs an engineered peptide ligase, localized to the cell surface either through exogenous overexpression of a plasma membrane-targeted fusion protein (384) or chemical functionalization for covalent attachment to abundant cell surface glycans (385). This ligase enzymatically labels accessible free N-termini of cell surface proteins with a peptide ester containing biotin and an isotopic tag, enabling specific capture and analysis of N-terminal peptides in an MS-based proteomic workflow. This approach was used to examine broad alterations in cell surface proteolysis following the induction of single oncogenes in premalignant cells (385). Future analyses using protease knockout models might connect specific cleaved cell surface substrates to particular protease mediators in various cancer contexts. A different useful method for labeling surface glycans for proteomics is *surface-spanning protein enrichment with click sugars* (SUSPECS), which has been used for the identification of substrates of several sheddases, albeit not yet in cancer models (386, 387).

Another innovative chemical biology approach for substrate identification employs protease engineering through genetic code expansion, creating *mechanism-based covalent traps* that can capture and identify the natural protein substrates of specific proteases (388). By replacing the catalytic serine or cysteine nucleophile of a serine or cysteine protease with genetically encoded, photocaged 2,3-diaminopropionic acid, the modified enzyme, upon photoactivation, can form a stable acyl-enzyme adduct with the N-terminal substrate fragment. These enzyme-substrate adducts can then be isolated for substrate identification using an MS workflow (388). While not yet widely adopted, this method holds immense potential for identifying comprehensive substrate repertoires for the many extracellular serine and cysteine proteases implicated in cancer processes.

Candidate substrates identified through broad screening approaches require subsequent validation to confirm whether the protease-substrate relationship is direct. The presence of a cleavage fragment in the presence of the protease might be due to indirect proteolysis promotion, such as when the protease activates another protease responsible for the cleavage or inactivates an inhibitor of another protease. Traditionally, validation studies have tested direct cleavage using purified recombinant proteins with gel-based readouts. However, current strategies also incorporate mass spectrometry-based *targeted proteomics* experiments to detect and quantify fragments produced by the cleavage of a specific substrate (380).

Multiplexing of samples over a time course for *time-resolved substrate degradomics* can reduce false positives and provide insight into cleavage kinetics. This approach allows for subclassification and prioritization of candidate substrates based on their catalytic efficiency of cleavage (389). Once a substrate is validated as genuine, further mechanistic studies in biological models may be required to understand the significance of the proteolytic cleavage event in the cancer context. These steps are critical for unraveling the complex interactions within the protease web and their implications for cancer progression and treatment.

### Probes and reagents for enzyme identification, detection, and disambiguation

In some studies, the goal is to identify the active proteases in specific cancer settings or to determine the proteases responsible for observed proteolytic activities in cancer. Since protease activity is regulated posttranslationally *via* zymogen latency and endogenous inhibitors, as detailed in the section, “*The making of villains – posttranslational regulation and dysregulation of proteolytic activity*”, simply monitoring protease abundance alone is misleading. Even quantification of the mature protease form is an insufficient proxy for activity, since mature proteases may be inactive due to inhibition. It is therefore necessary to directly detect or capture active proteases. *Activity-based protein profiling* (*ABPP*) is a technique that fulfills this need by using probes that bind and covalently label active forms of proteases, enabling their isolation and identification by proteomics methods (390, 391). This method is particularly effective for the serine and cysteine protease families since their chemical mechanisms feature a reactive serine or cysteine nucleophile that readily undergoes covalent labeling by an electrophilic probe (391). Seminal studies have used broadly reactive probes to profile serine hydrolases, including proteases, in cell-based breast cancer models (392) and in cells derived from mammary tumor xenografts (393). These studies found that increased invasiveness and metastatic propensity were associated with increases in uPA and tPA activity. For labeling metalloproteases, covalent probes have been developed using photoreactive or spontaneously reactive variants of noncovalent inhibitors (391, 394, 395). However, these approaches have not yet been widely adopted, partly due to challenges in achieving uniform labeling efficiencies across different enzymes. This limitation arises because the proximity labeling depends upon the presence of a well-placed nucleophile on the enzyme (395, 396).

Activity-based probes with selectivity for specific families or subsets of proteases have been used to identify key proteases involved in cancer malignancy. For instance, a probe specific to the cysteine cathepsin family was used to image cathepsin activity in the RIP1-Tag2 pancreatic tumorigenesis model. Isolation of labeled enzymes from tissue lysates revealed increased activities of cathepsins B, C, L, and Z during tumor progression (397). A recent study used an activity-based probe designed to broadly label serine proteases with tryptic-like specificity (those cleaving after lysine or arginine), identifying uPA and tPA as active serine proteases that promote malignant properties of ovarian clear cell carcinoma cells (398). Sometimes research needs to answer more specific questions, such as identifying the protease responsible for cleaving a specific substrate in a particular setting. To identify the primary protease responsible for cleaving CDCP1 on the tumor cell surface, given previous indications of involvement of serine proteases with tryptic-like specificity, *substrate-biased activity-based probes* were specially designed to mimic the two known relevant cleavage sites within CDCP1 (367). These probes successfully captured and identified tumor cell-secreted uPA as the major protease responsible for CDCP1 cleavage and consequent promotion of pancreatic cancer metastasis.

Significant efforts have been invested in creating highly specific activity-based probes for imaging and functional analyses of individual proteases in culture and *in vivo* models (399). These probes can be valuable for distinguishing closely related enzymes that share similar expression patterns and substrate profiles. An example is in the development of activity-based probes capable of discriminating between the very similar TTSPs matriptase and hepsin (400). Often, probes with improved specificity are developed by screening combinatorial peptide libraries that incorporate both natural and noncanonical amino acids (401). Examples include the development of orthogonal probes designed to differentiate among the four human neutrophil serine proteases, particularly the closely similar neutrophil elastase and proteinase 3 (402, 403).

A general approach that shows great promise for the identification of highly selective activity-based probes for serine or cysteine hydrolases employs phage display of cyclized peptides, derivatized with an appropriate reactive electrophile, to screen large diversity libraries of candidate probe sequences (404). A different approach to address the challenge of closely similar active sites among homologous enzymes resorts to disambiguation through enzyme engineering. By mutating the target enzyme to contain an additional, noncatalytic cysteine nucleophile near the active site, an appropriately matched activity-based probe can be rendered exquisitely selective (405). Through the knock-in of the engineered enzyme into appropriate cancer cell models, the cognate probe could be used to selectively image and quantify the active protease during various cancer-related processes. Importantly, the utility of this approach need not be limited to serine or cysteine proteases but might also be extended to metalloproteases or aspartic proteases.

The development of sensitive and selective activity-based probes for various KLKs has enabled detailed studies of active KLK6 in pancreatic cancer cells (406) and the simultaneous orthogonal detection of active KLK2, KLK3, and KLK14 in prostate cancer culture models (407). In the realm of cysteine proteases, selective activity-based probes have been developed for cathepsins B and L, showing promise for the detection and localization of these active proteases in cancer cells, including primary tumor cells isolated from patient samples (408, 409). These developments highlight the growing precision and utility of activity-based probes in cancer research, enhancing our understanding of protease functions in malignancy.

For some applications, *antibodies* and other *protein-based affinity reagents* can offer an alternative to covalent activity-based probes, harnessing the potential for specificity that can be gained through the large and complementary interface of a protein–protein interaction. For example, an active-site-specific recombinant antibody was used to selectively image active matriptase on the cancer cell surface, both in cultured tumor cells and in mouse models (410). Protein affinity reagents have been pursued for MMPs, where the development of selective covalent affinity-based probes has proven elusive. Libraries of single-chain *camelid antibodies*, and human antibodies engineered with convex paratopes resembling camelid antibodies, have proven to be rich sources for inhibitory antibodies that can selectively target the accessible active sites of individual MMPs, including MMP-14 and MMP-10, with high affinity (411, 412). Alternatively, natural *TIMPs* can be used as scaffolds to develop high affinity and specificity reagents targeting the active sites of individual MMPs. Variants of the N-terminal domain of TIMP-2 that can selectively target active MMP-14 or MMP-9 in cancer cell cultures have been identified by screening combinatorial libraries using the yeast surface display platform (413, 414, 415). Similarly, yeast surface display of a full-length TIMP-1 library enabled the identification of an MMP-3-selective variant capable of fine discrimination between the highly conserved MMP-3 and MMP-10 (416). These recent successes suggest that TIMP and antibody engineering may offer paths toward an expanding toolbox of selective metalloprotease affinity reagents to probe the distinct functions of these extracellular enzymes in biological models.

In addition to detecting active enzymes, affinity reagents can selectively identify *neo-epitopes* generated by proteolytic cleavage within crucial substrates. Monoclonal antibodies that specifically recognize these neo-epitopes are invaluable tools to elucidate the impact of the cleavage events in biological systems. A notable instance of this approach was used to study the cleavage of laminin-111 by neutrophil elastase and MMP-9 in association with NETs as a trigger for dormant cancer cell reactivation (108). A monoclonal antibody raised against NET-remodeled laminin-111 uniquely identified cleaved, but not intact, laminin. This antibody helped show that sites of laminin remodeling were aligned with NETs, that the cleavage was dependent on the presence of both neutrophils and NETs, and that the cleaved laminin-111 colocalized with tumor cell clusters undergoing reawakening. Additionally, chimeric mouse antibodies targeting the neoepitope blocked dormant tumor cell reawakening *in vitro* and in mice, providing strong evidence for the direct role of NET-induced proteolytic laminin cleavage in cancer recurrence (108).

Beyond mechanistic investigations, antibodies recognizing neo-epitopes have potential as clinical biomarkers or for pharmacological targeting. For example, a recent study developed recombinant antibodies capable of recognizing a tumor-specific proteolytic neoepitope of CDCP1 without binding to the uncleaved form (417). These neoepitope-targeting antibodies were then used to direct the localization of antibody-drug conjugates in a pancreatic cancer model. This targeting approach localized the drug to tumors and showed superior efficacy with reduced toxicity compared to a pan-CDCP1 targeting approach, improving the therapeutic index.

## Concluding remarks: untangling the extracellular protease web in cancer progression, toward effective therapeutic interventions

Decades of research have unequivocally established an extensive network of extracellular proteases as pivotal players in cancer. These enzymes, by cleaving a variety of substrates within the tumor microenvironment, significantly contribute to tumor progression and metastasis. However, due to the complexities in protease regulation and their overlapping function within the extracellular microenvironment, untangling this protease web is challenging, and effective therapeutic interventions have remained largely elusive. For example, MMP-inhibiting drugs were developed in the 1990s and showed great promise in preclinical studies, but culminated in clinical trials with broadly disappointing outcomes (418). The reasons for the failure of these drugs were multi-fold; contributing factors included poor specificity and dose-limiting toxicity of the drugs, incomplete understanding of the biology, and trial designs that were, in retrospect, nonideal. Treated patients were in very late stages of the disease, when many of the aspects of cancer progression fueled by MMPs had already occurred (418).

Over the past several years, agents developed to more selectively target individual proteases or smaller sub-families have been brought forward into clinical trials. Recent targets include MMP-9, MT1-MMP, sheddases ADAM-10 and ADAM-17, and cell-surface serine proteases DPP IV and FAP (298, 419). Agents include therapeutic antibodies as well as small molecules and in some cases more complex conjugates. Strategies for targeting cell surface-anchored proteases can extend beyond merely blocking proteolytic activity, and use them as markers to direct immunotherapies toward particular cell types or as activators for pro-drug conjugates (419). For example, recent efforts to target FAP stem from the observation that this protease is expressed nearly exclusively on tumor-promoting cancer-associated fibroblasts. This specific localization enables immunotherapeutic strategies such as chimeric antigen receptor (CAR)-T cells engineered to eliminate these tumor-promoting fibroblasts *via* recognition of FAP (419, 420). While this approach holds promise, in general, the outcomes from clinical trials of protease inhibitors in cancer have continued to show disappointing or mixed results, likely reflecting the complexity of the proteolytic landscape.

To address the challenge, we need both big-picture insights from a systems approach, and yet more detailed cataloging of the individual components and interactions within the system. Continued efforts to link specific proteases to their specific substrates and to connect distinct cleavage events to their pathological consequences are essential. This effort involves tracing the actions and impacts of individual molecular entities within the broader narrative of cancer. Integrating this knowledge across the myriad enzymes and families will help identify critical control points and potential targets for intervention. Furthermore, this endeavor requires a context-specific lens, acknowledging that the ensemble of key players varies across different types of cancer, stages of progression, and distinct tumor microenvironments. Encouragingly, the development of new tools and methodologies is facilitating these advances. Innovations in mass-spectrometry-based substrate profiling, activity-based probes, and other chemical biology tools are enhancing our ability to study proteases in more nuanced and sophisticated ways. These technological leaps hold the promise of not only deepening our understanding of cancer biology but also, at last, of introducing effective therapeutic interventions targeting proteases and proteolytic pathways.

## Conflict of interest

The author is an Editorial Board Member for the Journal of Biological Chemistry and was not involved in the editorial review or the decision to publish this article.
